# Possible Involvement of Lysophospholipids in Severe Asthma as Novel Lipid Mediators

**DOI:** 10.3390/biom15020182

**Published:** 2025-01-27

**Authors:** Hiroaki Kume, Kentaro Kazama, Riko Sato, Yuki Sato

**Affiliations:** Department of Infectious Diseases and Respiratory Medicine, Fukushima Medical University Aizu Medical Center, 21-2 Maeda, Tanisawa, Kawahigashi, Aizuwakamatsu 969-3492, Japan; kazama10@fmu.ac.jp (K.K.); riko-f@fmu.ac.jp (R.S.); yukisato@fmu.ac.jp (Y.S.)

**Keywords:** lysophosphatidic acid, lysophosphatidylserine, lysophosphatidylcholine, sphingosine 1-phosphate, epithelial cells, endothelial cells, smooth muscle cells, eosinophils, mast cells, allergic diseases, refractory asthma

## Abstract

In severe asthma, symptoms are unstable despite intensive treatment based on high doses of inhaled corticosteroids and on-demand use of oral corticosteroids. Although, recently, various biological agents related to Th2 cytokines have been added to intensive controller medications for severe asthma, a significant progress has not been observed in the management for symptoms (dyspnea, wheezing and cough). Medical treatment focused on Type 2 inflammation is probably insufficient to maintain good long-term management for severe asthma. Airway eosinophilia and decreased reversibility in forced expiratory volume in 1 second (FEV_1_) are listed as major predictors for exacerbation-prone asthma. However, it is generally considered that asthma is complex and heterogeneous. It is necessary to establish precision medicine using treatable traits based on a multidimensional approach related to asthma. Since phospholipids generate lysophospholipids and arachidonic acid by phospholipases, lysophospholipids can be associated with the pathogenesis of this disease via action on smooth muscle, endothelium, and epithelium in the airways. Lysophosphatidic acid (LPA), lysophosphatidylcholine (LPC), and sphingosine 1-phosphate (S1P) are increased in bronchoalveolar fluid after allergen challenge. LPA, LPC, and S1P recruit eosinophils to the lungs and cause β_2_-adrenergic desensitization. LAP and S1P cause contraction and hyperresponsiveness in airway smooth muscle. Moreover, lysophosphatidylserine and S1P are associated with the allergic reaction related to IgE/FcεRI in mast cells. Lysophospholipid action is probably comprised of corticosteroid resistance and is independent of Type 2 inflammation, and may be corelated with oxidative stress. Lysophospholipids may be a novel molecular target in advancing the management and treatment of asthma. This review discusses the clinical relevance of lysophospholipids in asthma.

## 1. Introduction

Phospholipids that constitute the cell membrane are classified as glycerophospholipids and sphingolipids. It is generally thought that phospholipase (PL) A2 converts glycerophospholipids to lysophospholipids and free fatty acids. Biologically active eicosanoids (prostaglandins and leukotrienes) are closely related to the pathophysiology of asthma. Platelet-activating factor (PAF), which is synthesized from lyoPAF by acetyltransferase, is involved in the inflammatory processes of asthma [1,2,3]. Moreover, it has recently been reported that other lysophospholipids are probably associated with the pathophysiology of this disease [4,5,6]. However, details are still unknown about it.

**Figure 1:**
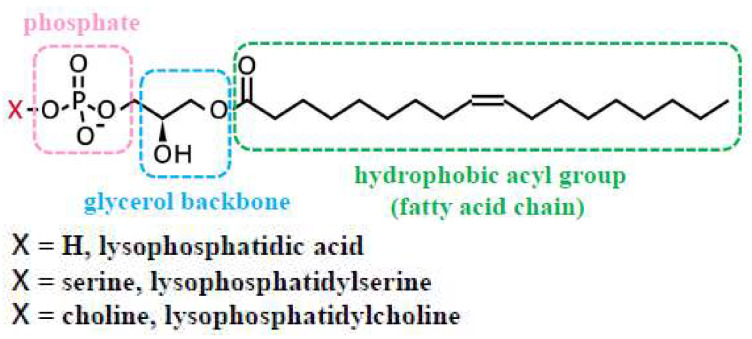
The representative structures of glycerophospholipids that consist of a glycerol backbone. The number of carbons and double bonds varies depending on each fatty acid.

**Figure 2:**
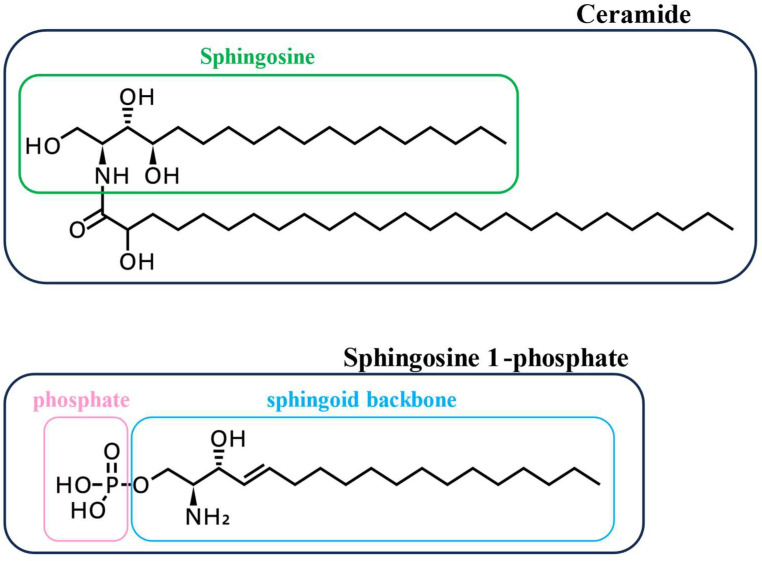
The representative structures of sphingolipids, the other form of phospholipid, are characterized by containing a sphingoid backbone. Sphingosine 1-phosphate, another type of lysophospholipid, is synthesized in the cell membrane from sphingomyelin through metabolites such as ceramide and sphingosine.

Lysophispholipids are synthesized from glycerophospholipids that consists of a glycerol backbone by phospholipases in the plasma membrane. They are released outside the cells. Lysophospholipids mainly contain lysophosphatidic acid (LPA), lysophosphatidylserine (LPS), lysophosphatidylcholine (LPC), etc. (Figure 1). Extracellular lysophospholipids act as lipid mediators and exert various physiological effects in inflammatory cells, such as mast cells, eosinophils and neutrophils [5,6,7,8,9,10] and airway constituent cells, such as epithelial [11], endothelial [12], and smooth muscle cells [13]. Sphingolipids, the other form of phospholipid, are characterized by containing a sphingoid backbone, such as sphingomyelin. Sphingosine 1-phosphate (S1P), another type of lysophospholipid, is synthesized in the cell membrane from sphingomyelin through metabolites, such as ceramide and sphingosine (Figure 2). S1P also acts as a lipid mediator by its release into extracellular fluids, and exerts various physiological effects in inflammatory cells, such as mast cells [14], eosinophils [15] and neutrophils [5], and airway constituent cells, such as epithelial [16], endothelial [17], and smooth muscle cells [18].

In severe asthma, symptoms (dyspnea, wheezing and cough) are unstable despite intensive treatment based on high doses of inhaled corticosteroids and on-demand use of oral corticosteroids. When various biological agents relate to IgE, thymic stromal lymphopoietin (TSLP) and Th2 cytokines (IL-4, IL-5, IL-13) are added to intensive controller medications; these agents are somewhat effective for a subgroup of patients with severe asthma. But their effects are limited, and significant progress has not been achieved in the management of this disease. Medical treatment focused on Type 2 inflammation, such as IgE, Th2 cytokines, and group 2 innate lymphoid cells (ILC2s) due to TSLP, is probably insufficient to maintain good long-term management (complete remission) for severe asthma. Airway eosinophilia and decreased reversibility in forced expiratory volume in 1 second (FEV_1_) are listed as major predictors for exacerbation-prone asthma [19,20,21]. However, asthma is complex and heterogenous. This disease is classified into phenotypes according to onset (juvenile and adult), allergy (atopy and non-atopy), and inflammation (eosinophil and non-eosinophil), including non-Type 2 inflammation. Serum IgE levels and inflammatory cytokines (IL-4, IL-5, IL-13) can link phenotypes with endotypes. However, the current standard therapy including these biological agents does not have the ability to allow for complete remission or cure asthma through overcoming airway hyperresponsiveness [22]. Although airway eosinophilia and airway hyperresponsiveness are the most important pathophysiologies in this disease, these mechanisms are not fully understood yet [23,24]. Therefore, it is necessary to establish precision medicine using treatable traits based on a multidimensional approach related to asthma.

Allergen challenges to patients with allergies increase lysophospholipids in the bronchial alveolar lavage fluid (BALF), indicating the involvement of lysophospholipids in allergic reactions, including asthma [4]. In this review, we explore the possible usefulness of lysophospholipids to improve therapy for severe asthma.

## 2. Lysophospholipids

### 2.1. Structures

Lysophospholipds (LPA, LPS, and LPC) are produced by PLA1 and PLA2 acting on glycerophospholipids, which include phosphatidic acid (PA), phosphatidylserine (PS), and phosphatidylcholine (PC) in the plasma membranes, respectively. Lysophospholipids are composed of a hydrophobic acyl group (one fatty acid chain with variations due to the carbon number ranging from 16 to 22 and an unsaturation degree of 0 to 6) and a glycerol backbone with a hydrophilic phosphate group. LPA, LPS, and LPC have a hydroxy group, serine, and choline attached to a glycerol backbone with a hydrophilic phosphate group, respectively (Figure 1).

**Figure 3:**
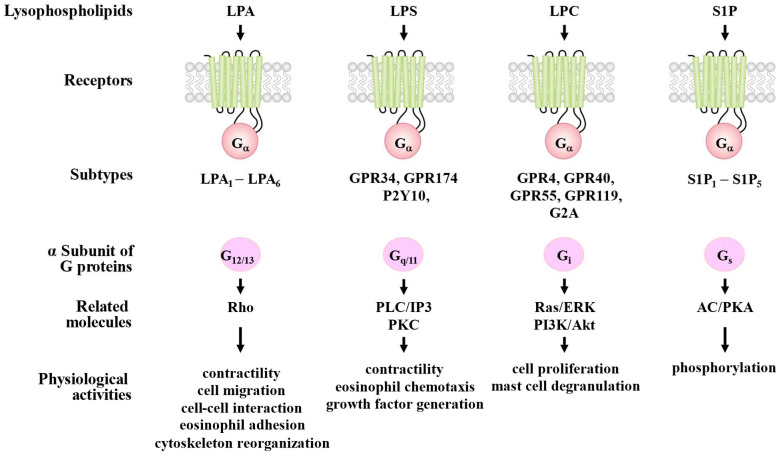
The receptors and intracellular signal transduction processes of lysophospholipids, and the physiological activities of lysophospholipids. LPA: lysophosphatidic acid; LPA_1_–LPA_6_: specifice receptors of LPA; S1P_1_–S1P_5_: specific receptors of S1P; LPS: lysophosphatidylserine; LPC: lysophosphatidylcholine; S1P: sphingosine 1-phosphate; GPR: G protein-coupled receptor; IP3: inositol 1,4,5-trisphosphate; PLC: phospholipase C; PKC: protein kinase C; AC: adenylyl cyclase; PKA: protein kinase A; ERK: extracellular signal-regulated kinase; PI3K: phosphoinositol 3-kinase; Akt: protein kinase B. Illustrated based on Refs. [5,26,27,28,29,30,31,32,33,35,36,37,38].

Extracellular lysophospholipids have various effects on many types of cells through their specific receptors belonging to G protein-coupled receptors (GPCRs) [5]. LPA binds to six subtypes of specific LPA receptors (LPA_1–6_), and LPA_1–3_ are endothelial differential gene (Edg_2,4,7_) types, respectively (Figure 3). On the other hand, LPA_4–6_ are non-Edg types. In addition to this, peroxisome proliferator-activated receptor gamma (PPARγ) is rgarded as an intracellular receptor of LPA [25]. LPS binds to three subtypes of specific LPS receptors (LPS_1–3_). G protein-coupled receptor 34 (GPR34), P2Y10, and GPR174 are currently shown to be specific receptors for LPS_1–3_, respectively [26,27,28] (Table 1). LPC also binds to the specific receptors. It is generally considered that GPR4, GPR40, GPR55, GPR119, G2A (GPR132), and Toll-like receptors, are probably specific receptors for LPC [29,30,31,32,33] (Figure 3). In addition to these, ovarian cancer G protein-coupled receptor 1 (OGR1), and T cell death-associated gene 8 (TDAG8), are thought to be specific receptors of LPC [33,34].

Sphingomyelin, the other phospholipid (sphingolipids), contains a sphingoid backbone, is metabolized to sphingosine through ceramide by sphingomyelinase and ceramidase (Figure 2), and sphingosine (the simplest shingolipids) is metabolized to S1P through phosphorylation by sphingosine kinase (SphK), which consists of SphK1 and SphK2. S1P is composed of sphingosine and a phosphate group (Figure 2). S1P binds to five subtypes of specific receptors (S1P_1–5_), which are GPCRs [5] (Figure 3). S1P_1–5_ belong to the Edg_1,5,3,6,8_ types, respectively.

### 2.2. Signal Pathways and Functions

The physiological activities of these lysophospholipids (LPA, LPC, LPS, S1P) are dependent on the α subunit of G proteins (G_α_), determining coupling with their specific receptors (Figure 3). Four types of the α subunit of G proteins (G_12/13_, G_q/11_, G_i_ and G_s_) are coupled to specific GPCRs for these lysophospholipids, and their effector signals are transduced in various cells, including inflammatory and airway constituent cells as described below [35,36] (Figure 3). G_12_/_13_ activates Rho/Rho-kinase processes, leading to contractility, migration, adhesion, cell–cell interaction, and cytoskeleton reorganization. G_q/11_ activates phospholipase C (PLC)/inositol 1,4,5-trisphosphate (IP_3_) processes, leading to contractility, chemotaxis, and growth factor generation (Figure 3). G_i_ activates Ras/extracellular signal-regulated kinase (ERK) processes and phosphoinositol 3-kinase (PI3K)/Akt processes, leading to cell proliferation (Figure 3). G_s_ activates adenylyl cyclase (AC)/protein kinase A (PKA) processes, leading to phosphorylation (Figure 3). Therefore, excessive lysophospholipids cause alteration in cell functions (phenotype changes) in lung cells. Furthermore, in the physiological activities of lysophospholipids (LPA_1_, GPR 34, and S1P_1_), the mechanisms of the conformational changes in receptors have recently been elucidated by an analysis of their cryo-electron microscopy structure [37,38].

## 3. Lysophosphatidic Acid

### 3.1. Structure and Function

An outline of the structure, signal pathways, and function of LPA is described in Section 2. The ligands recognized in LPA_6_ are associated with conformational rearrangement (inward shift in transmembrane helices 6 and 7) [39]. The activity of PLA1 or PLA2 converts PA to LPA in the cell membrane. As another pathway in intracellular synthesis, acylglycerol kinase (AGK) phosphorylates monoacylglycerol (MAG), leading to the production of LPA [40]. Moreover, LPA is synthesized from LPC by autotoxin (ATX) in the extracellular side. ATX, which is derived from the epithelium and macrophages, is a constitutively active enzyme possessing PLD activity, and is classified as ectonucleotide pyrophosphatase-phosphodiesterase 2 (ENPP2). LPA_1,2_ activate Rho/Rho-kinase, resulting in smooth muscle contraction via an increase in the response to intracellular Ca^2+^ (Ca^2+^ sensitization) [41,42] (Figure 3). Rho/Rho-kinase also leads to the dynamic reorganization of the actin cytoskeleton in the epithelium and endothelium [42], resulting in augmented cell migration and impaired cell–cell adhesion (Figure 3). In addition to this, LPA_1,2_ activate Ras/ERK and PI3K/Akt, resulting in cell proliferation (Figure 3). Therefore, LPA causes alteration in function (phenotype changes) in the lung cells [35]. LPA_1,2_ are probably potential target molecules for treating lung diseases. Recently, it has also been reported that LPA_5_ may be an important molecule for the establishment of advanced therapy against inflammation and malignancy [43].

### 3.2. Effects of Lysophosphatidic Acid on Airway Constituent Cells

#### 3.2.1. Epithelial Cells

The airway epithelium is the first tissue that contacts with inhaled microorganisms, allergens, and other environmental substances. Inflammatory cytokines are secreted from airway epithelial cells to modulate the immune response against inflammatory stimulation. Therefore, the airway epithelium plays an important part in various immune responses [44,45].

In bronchial epithelium, mRNA expression is abundant in the order LPA_1_, LPA_3_, LPA_2_, LPA_4_, and LPA_5_, and LPA_1–3_ exist in not only the plasma membrane, but also the cytoplasm of the cells [45,46]. LPA_1_ and LPA_2_ exist in the human airway epithelium [47]. Although the regulation of LPA receptor expression has not been sufficiently investigated, that expression may be varied between healthy individuals and patients with asthma. The expression of LPA_1_ mRNA may be upregulated approximately 3.2-fold; in contrast, that of LPA_2_ mRNA may be downregulated approximately 2.6-fold [45].

In the first step, external LPA increases the intracellular Ca^2+^ concentration and activates p38 mitogen-activated protein kinase (p38 MAPK), protein kinase C (PKC), and PLD, resulting in the exertion of physiological effects (immunity, cell proliferation, and barrier function) through the generation of cytokines and the activation of growth factors. LPA activates the nuclear factor (NF)-κB through p38 MAPK and PKCδ, resulting in increases in the secretion of interleukin (IL)-8, the expression of cyclo-oxygenase (COX)-2, and the release of prostaglandin (PG) E_2_ [48,49] (Table 1). LPA also increases the expression of the IL-13 receptorα2 (IL-13Rα2) without changing the mRNA levels of IL-4Rα [50] (Table 1). LPA also increases the expression of TSLP and the chemokine CCL20 via the regulation of NF-κB [51]. Therefore, LPA probably modulates immune responses, including the pathogenesis of asthma.

**Table 1:** The effects of lysophosphatidic acid on the constituent cells in the respiratory system, and related molecular mechanisms, physiological activities, and pathophysiology of asthma. PKC: protein kinase C; IL: interleukin; TSLP: thymic stromal lymphopoietin; COX-2: cyclo-oxygenase-2; PGE_2_: prostaglandin E_2_; EGF: epidermal growth factor; PDGF: platelet-derived growth factor; ICAM-1: intercellular adhesion molecule 1; VCAM-1: vascular cell adhesion molecule-1; MIP-1β: macrophage inflammatory protein-1β; MCP-1: monocyte chemoattractant protein-1.

Lysophosphatidic Acid				
Effetor Cells	Epithelial Cells	Endothelial Cells	Inflammatory Cells	Smooth Muscle Cells
Molecular mechanisms	PKCδ and ζ IL-8, IL-13, TSLP COX-2, PGE_2_, EGF, PDGF	Rho-kinase ICAM-1, VCAM-1 E-selectin, β1-integrin	Ca^2+^ entry MIP-1β, MCP-1, IL-8	Rho-kinase EGF
Biological activities	Cell proliferation Cell migration	Eosinophil adhesion Cytoskeleton reorganization	Mast cell degranulation Eosinophil chemotaxis Neutrophil chemotaxis	Contractility Cell proliferation Cell migration Cytoskeleton reorganization
Pathophysiology	Immune responses Airway remodeling	Barrier dysfunction Eosinophil recruitment	Eosinophil recruitment Neutrophil recruitment Allergic reactions	Airway hyperresponsiveness β_2_-adrenergic desensitization Airway remodeling
References	[40,47,48,49,50,51,52]	[6,53,54,55,56]	[57,58,59,60,61,62,63]	[64,65,66,67,68,69,70,71,72,73,74,75,76,77,78,79,80,81,82,83,84]

**Figure 4:**
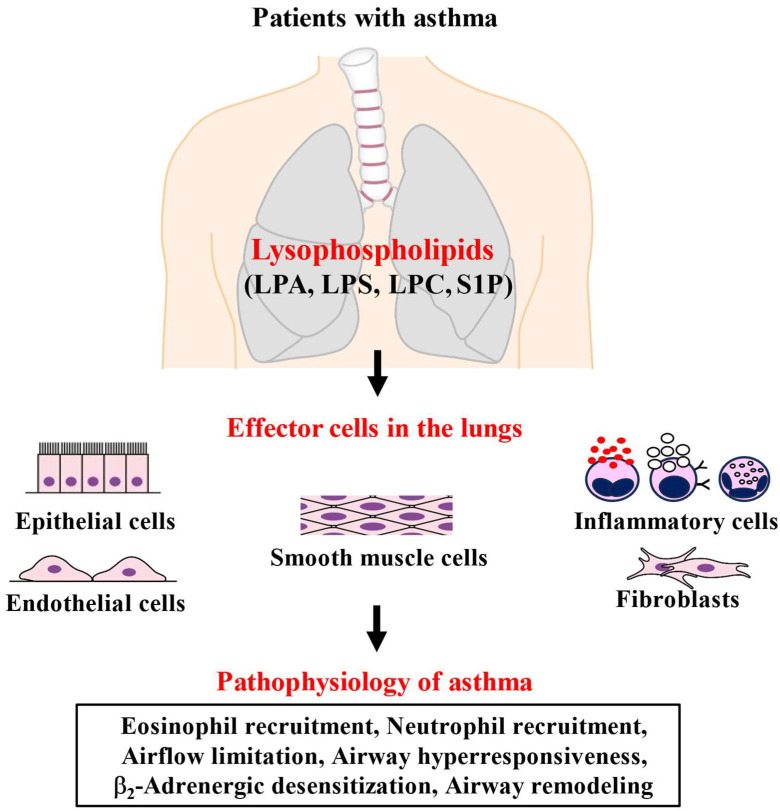
Involvement of lysophospholipids in the pathophysiology of asthma. LPA, LPC, and S1P are elevated in bronchial alveolar lavage fluids in patients with asthma, and LPS causes degranulation from eosinophils and mast cells. These lysophospholipids affect the pulmonary constituent cells. These phenomena are closely associated with the major pathophysiology of asthma. LPA: lysophosphatidic acid; LPS: lysophosphatidylserine; LPC: lysophosphatidylcholine; S1P: sphingosine 1-phosphate. Inflammatory cells: eosinophils, mast cells, and neutrophils. Illustrated based on Refs. [7,47,88,89,90,91,92].

LPA causes a transactivation (tyrosine phosphorylation) of epidermal growth factor receptor (EGFR) and the platelet-derived growth factor receptor b (PDGFRb) through PLD2 and PKCζ processes [40,49] (Table 1), leading to the regulation of gene expression and cell proliferation involving ERK1/2 and CCAAT/enhancer-binding protein β (C/EBPβ). The transactivation of EGFR induces IL-3 secretion, COX-2 expression, and IL-13Rα2 expression as the second pathway [49]. LPA also enhances epithelial cell migration, which is reversed through the inhibition of PKCδ [52] (Table 1), resulting in the airway remodeling related to asthma (Figure 4). Moreover, LPA enhances the serine phosphorylation of c-Met through the activation of PKCδ and ζ, and c-Mer is relocalized with E-cadherin from the cytoplasm to cell–cell interaction, resulting in the regulation of the airway epithelial barrier function [85]. Treatment with LPA causes integrity in cell–cell conjugation in the airway epithelium [45,86]. Since it is generally thought that the bronchial epithelial barrier in asthma is in a functional disorder, this defect may facilitate the passage of allergens and other agents into the airway tissue, leading to immune activation [87]. Therefore, this effect of LPA may protect against the invasion of allergens through the dysfunction of airway epithelial barrier integrity in patients with asthma. However, further investigation is needed to clarify the.

#### 3.2.2. Endothelial Cells

In the analysis of the LPA receptor subtype, LPA_2,6_ are mostly expressed in human pulmonary arterial endothelial cells (HPAECs) and human microvascular endothelial cells (HMVECs); in contrast, LPA_1,3,4_ are modestly expressed in HPAECs [53]. It is generally thought that LPA exerts the effects of barrier dysfunction and adhesion molecule expression on pulmonary endothelial cells [6]. The exposure of HPAECs to LPA impairs barrier function in a concentration-dependent manner mainly through LPA_6_ independent of LPA_1_ [53], and, in contrast, another report has demonstrated that AM966, an inhibitor of LPA_1_, reduces barrier function in human pulmonary microvascular endothelial cells [54] (Table 1). Cytoskeleton reorganization due to fiber stress through Rho/Rho-kinase is probably associated with the LPA-induced loss of barrier function as the cell-specific mechanism [6,42] (Table 1).

It is generally thought that LPA induces the adhesion properties of pulmonary endothelium. Exposure to LPA increases the expression of intracellular adhesion molecule 1 (ICAM-1), E-selectin, β1-integrin, and vascular cell adhesion molecule 1 (VCAM-1), which are adhesion molecules [55] (Table 1). These effects probably cause the infiltration of inflammatory cells to the lungs. When LPA is inhaled in guinea pigs, the number of neutrophils and eosinophils is increased in BALF in a concentration-dependent fashion, and, followed by that, more superoxide is generated [56] (Table 1). The LPA-induced infiltration of the inflammatory cell and LPA-induced superoxide synthesis are inhibited by Y-27632, a Rho-kinase inhibitor [56]. The Rho/Rho-kinase processes are associated with these phenomena related to LPA. LPA may be involved in airway eosinophilic inflammation in asthma (Figure 4).

#### 3.2.3. Inflammatory Cells

Since LPA_2_ is expressed in the high level in naïve CD^4+^, LPA facilitates chemotaxis but reduces IL-2 production [93]. On the other hand, since LPA_2_ is expressed at low levels in activated T lymphocytes, LPA reduces chemotactic responses but increases in IL-2 production. LPA can enhance IL-13 gene expression in T lymphocytes through synergy with other T cells [94], indicating involvement in Th2 inflammation.

Dendritic cells (DCs) are involved in antigen presentation in immune responses in mammals. The detailed effects of LPA on DCs are still unknown. Previous reports have suggested that LPA activates or inhibits DC functions [10]. However, the activation of LPA_2_ may cause an inhibitory regulation of DC activation and allergic inflammation [95].

LPA enhances monocyte migration [96]. In addition to this, LPA differentiates monocytes to macrophages through the activation of PPARγ, a non-canonical LPA receptor [97]. LPA synthesizes IL-1β and reactive oxygen species (ROS) in macrophages [98], indicating that this phenomenon may be associated with chronic inflammatory diseases [99].

Neutrophils are activated through LPA-induced Ca^2+^ dynamics [57]. LPA facilitates chemotactic responses on neutrophils [58], and it also facilitates migration through binding with LPA_1_ [59] (Table 1). LPA action on eosinophils is essentially similar to that on neutrophils. LPA has effects on chemotaxis and Ca^2+^ dynamics in human eosinophils [60] (Table 1). Moreover, LPA causes ROS production in eosinophils, similar to macrophages [60]. Inhalation of LPA causes the infiltration of neutrophils and eosinophils to the lungs in guinea pigs [56]. Mast cells are differentiated through exposure to LPA [61], and the release of histamine and tryptase is enhanced, and the generation of macrophage inflammatory protein (MIP)-1β, IL-8, and monocyte chemoattractant protein (MCP)-1 are augmented [62,63] (Table 1). Therefore, LPA is probably associated with inflammatory responses related to asthma and chronic obstructive pulmonary disease (COPD) (Figure 4).

#### 3.2.4. Smooth Muscle Cells

LPA does not have an effect on airway smooth muscle tone; however, LPA enhances muscarinic agonist-induced contraction [64]. In addition to this, LPA reduces β_2_-adrenergic agonist-induced relaxation [64]. LPA is probably involved in airway hyperresponsiveness and β_2_-adrenergic desensitization, which are related to the pathophysiology of asthma (Table 1). The functional antagonism between muscarinic and β_2_-adrenergic receptors probably converge on the G protein (G_i_, G_s_)/Ca^2+^-activated K^+^ (K_Ca_) channel pathway [65,66,67,68] and the RhoA/Rho-kinase pathway [42,69,70]. The former causes Ca^2+^ dynamics due to voltage-dependent Ca^2+^ channels [71,72], the latter causes Ca^2+^ sensitization due to myosin phosphatase [42,62,63]. The functional antagonism between these GPCRs is involved in airway hyperresponsiveness [18,73] and β_2_-adrenergic desensitization [13,74,75,76,77]. Ca^2+^ signaling due to these intracellular pathways is probably associated with cross talk (synergistic action) between muscarinic antagonists and β_2_-adrenergic agonists [72,78]. LPA does not cause contraction with Ca^2+^ entry; however, Y-27632, a Rho-kinase inhibitor, attenuates LPA-induced contractility under the experimental conditions of embedded collagen gel [79] (Table 1). Rho-kinase-induced Ca^2+^ sensitization probably causes the LPA-induced hyperresponsiveness to muscarinic agonists and LPA-induced hyporesponsiveness to β_2_-adrenergic agonists (Table 1).

LPA enhances cell proliferation through synergistic action with epidermal growth factor (EGF) for facilitating mitogenesis [80,81] (Table 1). The upregulation of EGF receptors and the activation of multiple transcription factors are probably associated with the mechanisms of this phenomenon [82]. LPA also causes the migration of bovine tracheal smooth muscle cells from the wounded confluent monolayer (Table 1); however, the chemotactic migration is not facilitated toward LPA, referred to as random migration [83]. This LPA-induced migration is inhibited by β_2_-adrenergic receptor agonists and other cAMP/protein kinase A-related agents (dibutyryl cAMP, forskolin) [83]. LPA causes actin cytoskeleton reorganization in the wound edge, but not in migrated cells, through RhoA/Rho-kinase processes, indicating that LPA-induced cell motility may play an important role in the initiation of cell migration [83] (Table 1). In contrast, previous reports have indicated that LPA causes actin cytoskeleton reorganization (augmented filamentous actin staining) through Rho-kinase [84]. LPA-induced actin cytoskeleton reorganization is inhibited in the presence of the C3 exoenzyme, an inactivator of RhoA, and GGTI-2147, an inhibitor of geranylgeranyltransferase (RhoA isoprenylation) [84,100]. Therefore, LPA has these effects on proliferation, migration, and actin cytoskeleton reorganization, resulting in airway remodeling related to the pathophysiology of asthma (Figure 4).

### 3.3. Involvement in Asthma

The physiological activities exerted by LPA are associated with the pathophysiology of asthma, such as mast cell degranulation, eosinophil recruitment, barrier dysfunction, β_2_-adrenergic receptor agonists, airway hyperresponsiveness, and airway remodeling (Table 1). LPA is probably involved in asthma by affecting the pulmonary constituent cells. including inflammatory cells [88,101,102] (Figure 4). A previous report demonstrated that LPA is detectable in BALF from patients who have allergic diseases, and that LPA is markedly increased in them following allergen challenges [47] (Figure 4). LPA levels are not significantly consistent with augmented eosinophils, neutrophils, or lymphocytes, suggesting that LPA causes barrier integrity by acting on airway epithelial cells. ATX and polyunsaturated LPA are markedly elevated in BLAF from patients with mild asthma, who are identified using an allergen skin test and a methacholine challenge test, after allergen challenges [88]. Moreover, eosinophils, IL-4, and IL-5 are more increased in BALF from the ATX transgenic mice after allergen challenges than that from wild-type mice [88]. In contrast, IL-4 and IL-5 levels are more attenuated in BALF from the LPA_2_ knock-out mice than that from wild-type mice [88]. H2L5186303, an antagonist of LPA_2_, suppresses increases in eosinophil counts and Th2 cytokine (IL-5 and IL-13) in BALF in a murine model of asthma after allergen challenges [103]. Airway hyperresponsiveness is generally defined as hyperresponsiveness to muscarinic agonists and histamine using measurement of reduced FEV_1_ or respiratory resistance by the cumulative inhalation of these agents. H2L5186303, an antagonist of LPA_2_, inhibits augmented respiratory resistance induced by the administration of methacholine, an agonist of muscarinic receptors, in the sensitized mice in measurements using restrained plethysmography [103]. In contrast, an agonist of LPA_2_, 2-[4-(1,3-dioxo-1H,3H-benzoisoquinolin-2-yl)butylsulfamoyl]benzoic acid (DBIBB), reduces Type 2 inflammation in the airways of allergen-challenged mice [104]. LPA_2_ may also inhibit DC function and allergic airway inflammation [10,95]. Although the ATX-LPA axis can be a potential target for the management and treatment of asthma [105], the role of LPA in asthma is not fully understood yet.

## 4. Lysophosphatidylserine

### 4.1. Structure and Function

An outline of the structure, signal pathways, and function of LPS is described in Section 2. A recent report shows the relationship between LPS receptors and the subunits of G protein. LPS_1_ (GPR34) is coupled to G_i_, LPS_2,3_ (P2Y10, GPR174) are coupled to G_12/13_, and LPS_3_ (GPR174) is also coupled to G_s_ [106] (Figure 3). It is well known that the plasma concentration of LPS is approximately 10 nM (much less than that of S1P), and this concentration is insufficient to activate these receptors, suggesting that LPS acts as a local lipid mediator, not a systemic lipid mediator. The most characterized physiological activities of LPS are an acceleration of degradation in inflammatory cells (mast cells and eosinophils) [7] (Table 2), indicating that LPS is involved in allergic reactions, including asthma. Analysis of the structure of GPR34 using cryo-electron microscopy may elucidate the mechanisms of endogenous agonist recognition and antagonist inhibition in this receptor, leading to a promising strategy for disease management [28].

### 4.2. Effects of Lysophosphatidylserine on Airway Constituent Cells

#### 4.2.1. Epithelial Cells

As a novel proinflammatory function of LPS, a recent report has indicated that LPS may induce Mucin 5 subtype AC (MUC5AC) production in airway epithelial cells through the positive feedback control of the tumor necrosis factor-alpha converting enzyme (TACE)-EGFR-ERK pathway, independent of receptors and ROS [107] (Table 2). It is generally thought that an excessive production of Mucin may lead to worsening condition in asthma and COPD. However, since anti-asthmatic agents that suppress mucin have never been developed, other than corticosteroids, this pathway may be a novel target molecule for therapy for asthma.

**Table 2:** The effects of lysophosphatidlyserine on the constituent cells in the respiratory system, and related molecular mechanisms, physiological activities, and pathophysiology of asthma. PI3K: phosphoinositol 3-kinase; ERK: extracellular signal-regulated kinase. MUC5AC: Mucin 5 subtype AC; PKC: protein kinase C; FcεRI: the high-affinity receptor for the Fc region of IgE.

Lysophosphatidylserine			
Effetor Cells	Epithelial Cells	Fibroblasts	Inflammatory Cells
molecular mechanisms	ERK	G_i_, PI3K, ERK PKC, Ca^2+^ entry	IgE/FcεRI
biological activities	MUC5AC production	Cell migration	Mast cell degranulation Eosinophil degranulation
pathophysiology	Airway inflammation	Airway remodeling	Allergic reaction
References	[107]	[107,108]	[7,26,88]

#### 4.2.2. Fibroblasts

LPS facilitates the chemotactic migration of fibroblasts, and this LPS-induced fibroblast migration is suppressed with an antagonist of ERK1/2 (PD98059) and a broad-spectrum antagonist of PI3K (LY294002) [108] (Table 2). PI3K and EFK are associated with this phenomenon [107]. Moreover, since LPS-induced fibroblast migration suppresses exposure to an inhibitor for coupling between G_i_ and GPCRs (pertussis toxin), G_i_ is associated with this phenomenon [107]. However, another report has indicated that LPS augments intracellular Ca^2+^ concentration through PKC, but is insensitive to pertussis toxin [108].

#### 4.2.3. Inflammatory Cells

LPS causes histamine release and eicosanoid production via the activation of GPR34 in mast cells [109] and promotes degranulation through the activation of P2Y10 in eosinophils [26] (Table 2). Furthermore, a previous report has demonstrated that LPS facilitates the antigen-induced cross-linking of IgE connected to high-affinity IgE receptors (FcεRI), resulting in an increase in histamine release from peritoneal mast cells [7]. Hence, LPS is probably involved in allergic reactions, including asthma (Table 2). Nerve growth factor also enhances mast cell activation through interaction with LPS [110]. Moreover, LPS acts on T cells and attenuates their proliferation and generation through the activation of GPR174 [111], indicating that LPS may also be associated with immune responses.

### 4.3. Involvement in Asthma

The physiological activities exerted by LPS are associated with the pathophysiology of asthma, such as IgE-induced allergic reaction and airway remodeling (Figure 4). As described above, LPS facilitates the degranulation of mast cells through the activation of the IgE/FcεRI process [7] (Table 2). Furthermore, eosinophils from patients with severe asthma shows greater eosinophil extracellular trap formation and degranulation by a high concentration of LPS (50 μM) than those from patients with non-severe asthma (Figure 4), accompanied by significant expression of surface P2Y10 (a subtype of LPS receptor) [89]. LPS is also involved in eosinophilic inflammation in patients with severe asthma. P2Y10 may be a novel therapeutic molecule for severe asthma. Therefore, LPS is probably associated with IgE-induced allergic reaction and eosinophil-induced airway inflammation (the major pathophysiology of this disease).

## 5. Lysophosphatidylcholine

### 5.1. Structure and Function

An outline of the structure, signal pathways, and function of LPC is described in Section 2. Extracellular LPC causes various inflammatory responses, such as the production of proinflammatory cytokines, the expression of adhesion molecules and growth factors [112,113,114], the facilitation of monocyte chemotaxis [8,115], and the induction of macrophage activities [116], resulting in the onset of atherosclerosis and other inflammatory diseases [32,117,118]. LPC activates eosinophils [9] and T-lymphocytes [119], and also augments the production of ROS, leading to the facilitation of oxidative stress [12]. Therefore, LPC is probably related to asthma and COPD [99]. These LPC-induced physiological effects are associated with intracellular Ca^2+^ signaling such as Ca^2+^ dynamics (Ca^2+^ entry and Ca^2+^ mobilization) [120] and Ca^2+^ sensitization (Rho-kinase activation) [13,121]. Molecules related to Ca^2+^ signaling can be therapeutic targets for these inflammatory respiratory diseases [32,122].

### 5.2. Effects of Lysophosphatidylcholine on Airway Constituent Cells

#### 5.2.1. Effects on Epithelial Cells

The exposure of vascular endothelial growth factor (VEGF) facilitates release for LPC from human bronchial epithelial cells, and released LPC has the ability to cause injury in the airway epithelium [123] (Table 3). In human ciliated epithelium, platelet-activating factor (PAF), lyso-PAF, and LPC cause reduced ciliary beating and damaged structural integrity through membrane-directed cytotoxic mechanisms which are induced by activated human polymorphonuclear leukocytes due to oxidative stress [124]. Therefore, barrier dysfunction is caused by LPC in the airway epithelium (Table 3). This phenomenon facilitates antigen entry, leading to the onset and exacerbation of allergic diseases including asthma. LPC-induced epithelia injury is suppressed with vitamin E, an antioxidant [124].

#### 5.2.2. Effects on Endothelial Cells and Inflammatory Cells

LPC induces the expression of chemokines, such as MCP-1 and IL-8, and is regulated on activation, and in normal T expression and secretion (RANTES), which cause chemotaxis in inflammatory cells, such as monocytes, in microvascular endothelial cells, and pertussis toxin (a Gi coupling inhibitor), and SB202190 (a p38 mitogen-activated protein kinase inhibitor) inhibits the LPC-enhanced expression of IL-8 and RANTES [125] (Table 3). It is generally thought that LPC in oxidized low-density lipoprotein (LDL) acts as a potent chemotactic factor for monocytes (recruitment of monocytes) [8], and, in addition to this, acts as a potent inhibitor of endothelial motility (limiting wound healing) [126]. LPC also has the ability to induce adhesion molecules in the endothelium, such as ICAM-1 and VCAM-1 [112], leading to the adhesion of monocytes to the endothelium (Table 3). Moreover, LPC facilitates migration and proliferation in smooth muscle cells and fibroblasts, resulting in the remodeling of the arterial walls through the expression of growth factors, such as platelet-derived growth factor (PDGF) and heparin-binding EGF-like protein (HB-EGF) [127]. LPC-induced adhesion molecules (ICAM-1 and VCAM-1) are associated with the G2A-related signaling pathway [114]. LPC is essentially involved in the pathophysiology of atherosclerosis. The CD11b/CD18 (Mac-1) molecule of the β2-integrin subfamily and CD49d/CD29 (VLA-4) of the β1-integrin subfamily causes the adhesion of eosinophils to endothelial cells through binding to ICAM-1 and VCAM-1 [128] (Table 3). LPC enhances the expression of CD11b/CD18 to induce eosinophil adhesion through Ca^2+^ entry due to a non-store-operated Ca^2+^ entry [129], resulting in eosinophil recruitment to the lungs in asthma [9]. GPR4 causes the upregulation of adhesion molecules in endothelial cells through the cAMP/PKA/cAMP pathway [130] (Table 3). Therefore, LPC causes eosinophil infiltration to the airway walls through the induction of adhesion molecules in endothelium in pulmonary vessels (Table 3).

#### 5.2.3. Effects on Smooth Muscle

In vascular smooth muscle, LPC (lysolecithin) probably impairs endothelium-dependent relaxation [131], and directly causes contraction in atherosclerosis [118]. On the other hand, although intracellular Ca^2+^ concentration is elevated with LPC in vascular smooth muscle with the endothelium removed, this LPC-induced Ca^2+^ entry does not cause contraction, but enhances the response to contractile agents [120]. The contractile response to angiotensin II is enhanced in the presence of LPS, and this effect is attenuated by Y-27632 [132]. Ca^2+^ sensitization via Rho-kinase activity is associated with physiological activities related to LPC.

In trachealis, LPC does not cause contraction without changes in Ca^2+^ levels inside the cells, and pre-treatment with LPC does not enhance the contractile response to methacholine (a muscarinic agonist) [13]. However, pre-treatment with LPC inhibits the relaxant response to isoproterenol (a β_2_-adrenergic agonist) against methacholine without changes in Ca^2+^ levels inside the cells [13] (Table 3). This desensitization to β_2_-adrenegic agonists is also reversed with Y-27632 [13], similar to tryptase-induced β_2_-adrenergic desensitization [77]. LPC reduces β_2_-adrenergic action through Rho-kinase-induced Ca^2+^ sensitization (Table 3, Figure 4). In contrast, Ca^2+^ entry due to the Ca^2+^-activated K^+^ channel/voltage-dependent Ca^2+^ channel linkage is involved in β_2_-adrenergic desensitization after excessive exposure to an agonist [71,74,75]. G_s_ function failure may be essential for reduced responsiveness to β_2_-adrenergic agonists [133]. The inhalation of LPC induces eosinophil infiltration to the lungs and causes a slow increase in respiratory resistance 6 h later [9]. This result indicates that LPC probably augments respiratory resistance via eosinophilic airway inflammation, not direct action on airway smooth muscle cells. It is still unknown whether LPC acts on proliferation and migration in airway smooth muscle cells, which are different from vascular smooth muscle cells [6].

### 5.3. Involvement in Asthma

The physiological activities exerted by LPC are associated with the pathophysiology of asthma, such as airflow limitation, eosinophil recruitment, barrier dysfunction, and β_2_-adrenergic desensitization (Table 3). Concentrations of LPC in BALF are elevated after mice are challenged with allergens [134]. Respiratory resistance is elevated after eosinophils are recruited to the respiratory system by the inhalation of LPC [9] (Table 3). The pre-exposure of LPC to tracheal smooth muscle causes modest effects on methacholine-induced contraction using isometric tension records in vitro [13]; however, the pre-inhalation of LPC enhances methacholine-induced respiratory resistance in guinea pigs in vivo [135]. LPC levels in BALF are also elevated with PLA2 activity in patients with asthma, compared to healthy individuals [90] (Figure 4). The plasma concentration of LPC is significantly raised in asthma and rhinitis compared with healthy individuals, and increased LPC levels are correlated with increased airway responsiveness to histamine in most cases of asthma [136] (Figure 4). These results indicate that LPC may be involved in airway contraction (airway limitation), eosinophil infiltration to the lungs (airway eosinophilic inflammation), and hyperresponsiveness to methacholine (airway hyperresponsiveness), which are essential features of asthma [137] (Table 3, Figure 4). Furthermore, LPC may induce emphysema, since LPC causes the dysfunction of pulmonary alveolar and epithelial cells through the enhancement of permeability and apoptosis [138,139]. Although LPC may be involved in the pathophysiology of asthma, it is still unclear which subtypes of LPC can be molecular targets for the management and treatment of this disease.

## 6. Sphingosine 1-Phosphate

### 6.1. Structure and Function

An outline of the structure, signal pathways, and function of LPA is described in Section 2. S1P_1_ is coupled with the inhibitory G protein of adenylyl cyclase (G_i_) in endothelial cells, leading to the activation of the Ras/ERK and PI3K/Ark processes (Figure 3) [140]. Although S1P_2,3_ are connected with multiple G proteins, S1P _2_ is mainly coupled with G_12/13_, leading to Ca^2+^ sensitization due to Rho-kinase activation, and S1P_3_ is mainly connected to G_q/11_, leading to Ca^2+^ entry due to PKC activation (Figure 3) [141]. S1P exhibits various physiological activities (proliferation, migration, differentiation, and actin cytoskeleton reorganization) on epithelium, endothelium, and smooth muscle cells in the respiratory system [142] (Figure 3). Moreover, S1P is probably involved in vascular development and function [143,144]. Since S1P is released in large quantities from various tissues [145], a higher concentration of S1P (~1 μM) is maintained in plasma in healthy subjects [145,146,147]; on the other hand, lower concentrations of S1P (~nM) are generally found in interstitial fluid [147]. Therefore, S1P in the plasma has the ability to exert various physiological activities on the respiratory system [148].

### 6.2. Effects of Sphingpsine 1-Phosphate on Airway Constituent Cells

#### 6.2.1. Effects on Endothelial and Inflammatory Cells

It is generally thought that S1P in plasma reduces vascular leakage through the integrity of the barrier function, and this physiological action is caused by S1P_1_/G_i_ and the Rac-induced recruitment of actin filaments [143,149]. A loss of S1P_1_ in the endothelium increases pulmonary vascular leakage [150], indicating that S1P_1_ probably has the ability to maintain the barrier function in the endothelium. Therefore, S1P probably suppresses acute respiratory distress syndrome (ARDS), including acute lung injury (ALI) that causes non-cardiogenic pulmonary edema due to pulmonary vasculature injury [147]. Since severe COVID-19 (severe acute respiratory syndrome coronavirus 2 infection) causes ARDS, S1P may also be a protective agent for severe cases of COVID-19 [151]. Hence, lower concentrations of S1P in the plasma can worsen prognosis in severe cases of COVID-19 [152]. A deficiency of SphK_1_ and SphK_2_ significantly reduces the concentration of S1P in the plasma and enhances vascular leakage in response to serotonin and histamine [149]. S1P_2_-deficient mice also have delayed histamine clearance [153] and lower resistance (higher mortality rate) to these anaphylaxis-induced agents than wild-type mice [149]. The expression of S1P_1_ is attenuated in pulmonary endothelium isolated from chronic smokers compared to non-smokers [154]. This phenomenon is correlated with the impairment of the autophagic response to S1P. S1P transiently augments intracellular Ca^2+^ levels through stretch-induced Ca^2+^ entry in the endothelium [155].

It is generally thought that S1P levels through SphK1 activation are augmented by the antigen/IgE/FcεRI pathway in mast cells, leading to the facilitation of degranulation [156,157], and this phenomenon is associated with allergic reactions, including asthma (Table 4). S1P has the ability to facilitate chemotaxis and the recruitment of eosinophils to the lungs through its receptors and CC chemokine receptor 3 (CCR3) [15,158]; moreover, external S1P also enhances the expression of VCAM-1 and ICAM-1 in the endothelium [15,159] (Table 4). S1P/Gi leads to the expression of these adhesion molecules, and this physiological activity is associated with Ca^2+^ dynamics through non-selective cation channels in HUVECs [160] (Table 4). However, the S1P-induced expression of ICAM-1 is modest in human pulmonary microvascular endothelial cells (HPMVECs) compared to that of VCAM-1 [15]. Pre-treatment with pertussis toxin and Y-27632 suppresses the S1P-induced expression of VCAM-1 in a concentration-dependent fashion [15] (Table 4). In an adhesion assay, S1P causes a concentration-dependent increase in the eosinophil adhesion to HPMVECs [15] (Table 4). S1P-induced eosinophil adhesion is reduced by pre-treatment with pertussis toxin and Y-27632 [15], indicating that G_i_ and Rho-kinase are probably associated with S1P action on the endothelium (Table 4). Moreover, S1P also has the ability to facilitate neutrophil chemotaxis, and to enhance lipopolysaccharide-induced chemotaxis in mice [161].

#### 6.2.2. Effects of Smooth Muscle

S1P generates tension in airway smooth muscle with increasing intracellular Ca^2+^ levels [18]. S1P-produced tension is attenuated with SKF-96364 (a Ca^2+^ channel inhibitor) and verapamil (a L-type voltage-dependent Ca^2+^ channel inhibitor) with decreased intracellular Ca^2+^ levels; in contrast, Y-27632 (a Rho-kinase inhibitor) suppresses it with no change in intracellular Ca^2+^ levels [18] (Table 4). Hence, S1P-induced contraction is caused by both Ca^2+^ dynamics and Ca^2+^ sensitization. S1P-induced contraction has also been observed in a collagen gel contraction assay using human and rat tracheal smooth muscle cells [162,163].

S1P augments the muscarinic contraction of airway smooth muscle with no change in intracellular Ca^2+^ levels, and this augmented muscarinic action independent of intracellular Ca^2+^ levels return to control levels with Y-27632 [18] (Table 4). Pre-treatment with pertussis toxin also suppresses this S1P-induced augmentation in muscarinic contraction [18] (Table 4). In human airway smooth muscle, pre-exposure to S1P causes increased reactivity to methacholine and histamine [164]. Systemic administration of S1P to mice also causes muscarinic hyperresponsiveness in the isolated bronchial tissues and total lungs [165]. Furthermore, external S1P probably causes heterologous β_2_-adrenergic desensitization in airway smooth muscle [166,167], similar to TGF-β_1_ [168] and PDGF [76]. Pre-exposure to S1P suppresses the relaxant action of isoproterenol and forskolin (a direct activator of adenylyl cyclase) without changes in the Ca^2+^ levels inside the cells, and this S1P-induced tachyphylaxis in these agents is returned to control levels with Y-27632 and pertussis toxin [166] (Table 4). These results indicate that external S1P is probably associated with airway hyperresponsiveness and β_2_-adrenergic desensitization through the intracellular signal pathway due to G_i_ and Rho-kinase (Table 4, Figure 4).

S1P facilitates the expression of forkhead box M1 (FOXM1) and cyclin D1 via yes-associated protein (YAP) dephosphorylation and nuclear localization, resulting in increases in proliferation and migration in rat tracheal and main bronchial smooth muscle cells, and this S1P-induced action is abolished with JTE013 (an antagonist of S1P_2_), CAY10444 (an antagonists of S1P_3_), and Y-27632 [163] (Table 4). S1P_2,3_ causes the proliferation and migration of airway smooth muscle cells, and Rho-kinase activity is related to this S1P action. S1P also causes a concentration-dependent increase in the cell proliferation of human airway smooth muscle through S1P_2_ [165] (Table 4). S1P probably leads to airway remodeling (Table 4, Figure 4).

### 6.3. Involvement in Asthma

The physiological activities exerted by S1P are associated with the pathophysiology of asthma, such as airflow limitation, mast cell degranulation, eosinophil recruitment, β_2_-adrenergic desensitization, airway hyperresponsiveness, and airway remodeling (Table 4). S1P-induced action is involved in asthma [14,16,91,169,170,171]. Allergen challenges to patients with asthma cause an increase in S1P concentration in BALF in these patients [172] (Figure 4). The administration of S1P to mice in the absence of allergens facilitates mast cell infiltration and airway hyperresponsiveness, and moreover, S1P increases IgE levels in plasma and Th2 cytokines (IL-4, IL-13) in lung tissues [173]. The S1P_2_ antagonist JTE-013 decreases eosinophil counts and Th2 cytokine (IL-4, IL-5) levels in BALF in allergen-challenged mice (animal model for asthma) [92]. S1P evokes action potential generation in afferent C-fibers of the vagal nerve in the airways, which is associated with wheezing and airway hyperreactivity; this S1P-induced action is suppressed with TY 52156 (a S1P_3_ antagonist) [174]. Since airway hyperresponsiveness is not observed in S1P_3_-deficient mice, S1P_3_ receptor antagonists may bring a novel therapeutic strategy for inhibiting airway hyperresponsiveness, which is related to asthma severity [175]. Therapy for airway hyperresponsiveness in asthma has not been established yet. SK1-I (a SphK1 inhibitor) reduces airway eosinophilia and airway hyperresponsiveness via the suppression of NF-κB after allergen challenge [176]. Furthermore, S1P generates tension and augments the response to muscarinic agonists in the airways [12,175]. S1P_2,3_ also facilitate smooth muscle proliferation and airway pro-remodeling action by Ca^2+^ signaling, including Rho-kinase activity, and this S1P-induced action is steroid-resistant [177]. Therefore, S1P is related to major features of asthma (airway obstruction, airway eosinophilia, airway hyperresponsiveness, airway remodeling, and β_2_-adrenergic desensitization) (Figure 4). Moreover, S1P_2_,_3_ may be novel therapeutic molecules for this disease.

In genetic analysis, genome-wide association studies (GWASs) show that the ORM (yeast)-like protein isoform 3 (ORMDL3) gene encoded in the 17q21 locus is probably associated with severity and exacerbations in asthma [178,179,180]. Allergen-induced airway neutrophilic inflammation is markedly attenuated with a decrease in IL-17 with reduced external S1P in the ORMDL3 transgenic mice compared to wild-type mice [161]. ORMDL3 overexpression reduces the biosynthesis of sphingolipids via the inhibition of serine palmitoyltransferase, leading to a decrease in the external level of S1P [161,181]. S1P may be involved in non-Th2 asthma due to airway neutrophilia. However, the impaired generation of sphingolipids due to ORMDL3 overexpression is associated with asthma exacerbation [182,183] and airway hyperresponsiveness [184]. Little detail is currently known about the involvement of S1P in non-Th2 asthma. Recently, it has been indicated that the elongation of very-long-chain fatty acid protein 6 (ELOVL6), a regulating enzyme for the elongation of saturated and monounsaturated fatty acids with C12 to C16 and those with C18, may be involved in asthma. The expression of ELOVL6 is enhanced in the bronchial epithelial cells of patients with severe asthma, and both Th2 and non-Th2 inflammation is enhanced with increases in ceramides and S1P in ELOVL6-deficiency mice, suggesting that ELOVL6 may be a novel target molecule for the treatment of asthma [185,186].

## 7. Conclusions

Since recent clinical trials have indicated that lysophospholipids are associated with patients with asthma [47,89,90,175] (Figure 4), lysophospholipids are probably novel target molecules for the development of asthma therapy [5,6,187]. Although lysophospholipids are generated with eicosanoids from phospholipids by PLA_2_, it is still unknown whether lysophospholipids are related to asthma specifically, rather than eicosanoids. However, since PLA_2_ group regulates immune responses against allergens, lysophopholipids may be associated with the pathogenesis of this disease [188]. Since lysophospholipids probably act as lipid mediators in asthma, they may be released from inflammatory cells and have an effect on epithelial, endothelial, and smooth muscle cells in the airways, similarly to chemical mediators. However, their origin is still unknown because lysophospolipids are probably generated in various tissues other than inflammatory cells in the airways.

Lysophospholipids act on GPCRs (muscarinic, β_2_-adrenergic receptor) and G protein (G_i_, G_s_, G_q/11_, G_12/13_) in the respiratory constituent tissues, such as epithelial, endothelial, smooth muscle, and inflammatory cells (mast cells, eosinophils, neutrophils), as described in the text. In these GPCRs, G proteins in the airway tissues are related factors in the onset of this disease. Moreover, the specific receptors of lysophospholipids are widely distributed in these inflammatory and respiratory constituent cells [4]. However, the effects of lysophospholipids on them have not be considered as molecular targets for precision medicine against asthma. Therefore, research on lysophospholipids may be beneficial for establishing treatment for the complete remission of asthma.

## Figures and Tables

**Table 3:** The effects of lysophosphatidlycholine on the constituent cells in the respiratory system, and related molecular mechanisms, physiological activities, and pathophysiology of asthma. VDGF: vascular endothelial growth factor; MAPK: mitogen-activating protein kinase; PKA: protein kinase A; MCP-1: monocyte chemoattractant protein-1; RANTES: regulated on activation, normal T expressed and secreted is the same as C-C motif chemokine ligand 5 (CCL5); IL: interleukin; ICAM-1: intercellular adhesion molecule 1; VCAM-1: vascular cell adhesion molecule-1.

Lysophosphatidlycholine			
Effetor Cells	Epithelial Cells	Endothelial Cells	Smooth Muscle Cells
Molecular mechanisms	VEGF	Gi, MAPK, PKA Rho-kinase, Ca^2+^ entry MCP-1, IL-8 RANTES, β2-integrin ICAM-1, VCAM-1	Rho-kinase
Biological activities	Ciliary beating disorder Structural integrity loss	Eosinophil adhesion	Contractility Respiratory resistance
Pathophysiology	Barrier dysfunction	Eosinophil recruitment	β_2_-adrenergic desensitization Airflow limitation
References	[123,124]	[112,114,125,126,127,128,129,130]	[9,13,120,131,132]

**Table 4:** The effects of sphingosine 1-phosphate on the constituent cells in the respiratory system and related molecular mechanisms, physiological activities, and pathophysiology of asthma. ICAM-1: intercellular adhesion molecule 1; VCAM-1: vascular cell adhesion molecule-1. FcεRI: the high-affinity receptor for the Fc region of IgE. RANTES: Regulated on activation, normal T cell expressed and secreted; CCR3: CC chemokine receptor 3; YAP: yes-associated protein.

Sphingosine 1-Phosphate			
Effetor Cells	Endothelial Cells	Inflammatory Cells	Smooth Muscle Cells
Molecular mechanisms	G_i_, Rho-kinase Ca^2+^ entry ICAM-1, VCAM-1	IgE/FceRI CCR3 RANTES	G_i_, Rho-kinase Ca^2+^ entry YAP
Biological activities	Eosinophil adhesion	Eosinophil chemotaxis Neutrophil chemotaxis Mast cell degranulation	Contractility Cell proliferation Cell migration
Pathophysiology	Eosinophil recruitment	Eosinophil recruitment Neutrophil recruitment Allergic reaction	Airflow limitation Airway hyperresponsiveness β_2_-adrenergic desensitization Airway remodeling
references	[15,155,159,160]	[15,156,157,158,161]	[18,162,163,164,165,166,167]

## Data Availability

Not applicable.

## References

[1] Bowton D.L., Seeds M.C., Fasano M.B., Goldsmith B., Bass D.A. (1997). Phospholipase A2 and arachidonate increase in bronchoalveolar lavage fluid after inhaled antigen challenge in asthmatics. Am. J. Respir. Crit. Care Med..

[2] Uozumi N., Kume K., Nagase T., Nakatani N., Ishii S., Tashiro F., Komagata Y., Maki K., Ikuta K., Ouchi Y. (1997). Role of cytosolic phospholipase A2 in allergic response and parturition. Nature.

[3] Nagase T., Ishii S., Shindou H., Ouchi Y., Shimizu T. (2002). Airway hyperresponsiveness in transgenic mice overexpressing platelet activating factor receptor is mediated by an atropine-sensitive pathway. Am. J. Respir. Crit. Care Med..

[4] Walsh M.T., Costello R. (2007). Putting fat on the fire? Lysophospholipid mediators in bronchoalveolar lavage fluid after allergen challenge. Clin. Exp. Allergy.

[5] Zhao J., Zhao Y. (2021). Lysophospholipids in Lung Inflammatory Diseases. Adv. Exp. Med. Biol..

[6] Kume H., Harigane R., Rikimaru M. (2024). Involvement of Lysophospholipids in Pulmonary Vascular Functions and Diseases. Biomedicines.

[7] Martin T.W., Lagunoff D. (1979). Interactions of lysophospholipids and mast cells. Nature.

[8] Quinn M.T., Parthasarathy S., Steinberg D. (1988). Lysophosphatidylcholine: A chemotactic factor for human monocytes and its potential role in atherogenesis. Proc. Natl. Acad. Sci. USA.

[9] Nishiyama O., Kume H., Kondo M., Ito Y., Ito M., Yamaki K. (2004). Role of lysophosphatidylcholine in eosinophil infiltration and resistance in airways. Clin. Exp. Pharmacol. Physiol..

[10] Kim S.J., Moon H.G., Park G.Y. (2020). The roles of autotaxin/lysophosphatidic acid in immune regulation and asthma. Biochim. Biophys. Acta Mol. Cell Biol. Lipids.

[11] Kassel K.M., Schulte N.A., Parker S.M., Lanik A.D., Toews M.L. (2007). Lysophosphatidic acid decreases epidermal growth factor receptor binding in airway epithelial cells. J. Pharmacol. Exp. Ther..

[12] Wolfram Kuhlmann C.R., Wiebke Lüdders D., Schaefer C.A., Kerstin Most A., Backenköhler U., Neumann T., Tillmanns H., Erdogan A. (2004). Lysophosphatidylcholine-induced modulation of Ca^2+^-activated K^+^ channels contributes to ROS-dependent proliferation of cultured human endothelial cells. J. Mol. Cell. Cardiol..

[13] Kume H., Ito S., Ito Y., Yamaki K. (2001). Role of lysophosphatidylcholine in the desensitization of β-adrenergic receptors by Ca^2+^ sensitization in tracheal smooth muscle. Am. J. Respir. Cell Mol. Biol..

[14] Oskeritzian C.A., Milstien S., Spiegel S. (2007). Sphingosine-1-phosphate in allergic responses, asthma and anaphylaxis. Pharmacol. Ther..

[15] Sashio T., Kume H., Takeda N., Asano T., Tsuji S., Kondo M., Hasegawa Y., Shimokata K. (2012). Possible Involvement of Sphingosine-1-Phosphate/G(i)/RhoA pathways in adherence of eosinophils to pulmonary endothelium. Allergol. Int..

[16] Sudhadevi T., Ackerman S.J., Jafri A., Basa P., Ha A.W., Natarajan V., Harijith A. (2024). Sphingosine kinase 1-specific inhibitor PF543 reduces goblet cell metaplasia of bronchial epithelium in an acute asthma model. Am. J. Physiol. Lung Cell. Mol. Physiol..

[17] Dudek S.M., Jacobson J.R., Chiang E.T., Birukov K.G., Wang P., Zhan X., Garcia J.G. (2004). Pulmonary endothelial cell barrier enhancement by sphingosine 1-phosphate: Roles for cortactin and myosin light chain kinase. J. Biol. Chem..

[18] Kume H., Takeda N., Oguma T., Ito S., Kondo M., Ito Y., Shimokata K. (2007). Sphingosine 1-phosphate causes airway hyper-reactivity by Rho-mediated myosin phosphatase inactivation. J. Pharmacol. Exp. Ther..

[19] Denlinger L.C., Phillips B.R., Ramratnam S., Ross K., Bhakta N.R., Cardet J.C., Castro M., Peters S.P., Phipatanakul W., Aujla S. (2017). Inflammatory and Comorbid Features of Patients with Severe Asthma and Frequent Exacerbations. Am. J. Respir. Crit. Care Med..

[20] Denlinger L.C., Heymann P., Lutter R., Gern J.E. (2020). Exacerbation-Prone Asthma. J. Allergy Clin. Immunol. Pract..

[21] Kume H., Watanabe N., Suzuki Y., Aslanidis T., Bersot C.D.A. (2023). Airway Disorders as Predictive Factors of Exacerbations in Asthma and COPD. Airway Management in Emergency Medicine.

[22] Leckie M.J., ten Brinke A., Khan J., Diamant Z., O’Connor B.J., Walls C.M., Mathur A.K., Cowley H.C., Chung K.F., Djukanovic R. (2000). Effects of an interleukin-5 blocking monoclonal antibody on eosinophils, airway hyper-responsiveness, and the late asthmatic response. Lancet.

[23] Chapman D.G., Irvin C.G. (2015). Mechanisms of airway hyper-responsiveness in asthma: The past, present and yet to come. Clin. Exp. Allergy.

[24] Barnig C., Frossard N., Levy B.D. (2018). Towards targeting resolution pathways of airway inflammation in asthma. Pharmacol. Ther..

[25] McIntyre T.M., Pontsler A.V., Silva A.R., St Hilaire A., Xu Y., Hinshaw J.C., Zimmerman G.A., Hama K., Aoki J., Arai H. (2003). Identification of an intracellular receptor for lysophosphatidic acid (LPA): LPA is a transcellular PPARgamma agonist. Proc. Natl. Acad. Sci. USA.

[26] Hwang S.M., Kim H.J., Kim S.M., Jung Y., Park S.W., Chung I.Y. (2018). Lysophosphatidylserine receptor P2Y10: A G protein-coupled receptor that mediates eosinophil degranulation. Clin. Exp. Allergy.

[27] Liang J., Inoue A., Ikuta T., Xia R., Wang N., Kawakami K., Xu Z., Qian Y., Zhu X., Zhang A. (2023). Structural basis of lysophosphatidylserine receptor GPR174 ligand recognition and activation. Nat. Commun..

[28] Xia A., Yong X., Zhang C., Lin G., Jia G., Zhao C., Wang X., Hao Y., Wang Y., Zhou P. (2023). Cryo-EM structures of human GPR34 enable the identification of selective antagonists. Proc. Natl. Acad. Sci. USA.

[29] Kabarowski J.H., Feramisco J.D., Le L.Q., Gu J.L., Luoh S.W., Simon M.I., Witte O.N. (2000). Direct genetic demonstration of G alpha 13 coupling to the orphan G protein-coupled receptor G2A leading to RhoA-dependent actin rearrangement. Proc. Natl. Acad. Sci. USA.

[30] Radu C.G., Yang L.V., Riedinger M., Au M., Witte O.N. (2004). T cell chemotaxis to lysophosphatidylcholine through the G2A receptor. Proc. Natl. Acad. Sci. USA.

[31] Liu P., Zhu W., Chen C., Yan B., Zhu L., Chen X., Peng C. (2020). The mechanisms of lysophosphatidylcholine in the development of diseases. Life Sci..

[32] Drzazga A., Okulus M., Rychlicka M., Biegała Ł., Gliszczyńska A., Gendaszewska-Darmach E. (2020). Lysophosphatidylcholine Containing Anisic Acid Is Able to Stimulate Insulin Secretion Targeting G Protein Coupled Receptors. Nutrients.

[33] Ludwig M.G., Vanek M., Guerini D., Gasser J.A., Jones C.E., Junker U., Hofstetter H., Wolf R.M., Seuwen K. (2003). Proton-sensing G-protein-coupled receptors. Nature.

[34] Xu Y. (2002). Sphingosylphosphorylcholine and lysophosphatidylcholine: G protein-coupled receptors and receptor-mediated signal transduction. Biochim. Biophys. Acta.

[35] Geraldo L.H.M., Spohr T.C.L.S., Amaral R.F.D., Fonseca A.C.C.D., Garcia C., Mendes F.A., Freitas C., dosSantos M.F., Lima F.R.S. (2021). Role of lysophosphatidic acid and its receptors in health and disease: Novel therapeutic strategies. Signal Transduct. Target. Ther..

[36] Nagahashi M., Takabe K., Terracina K.P., Soma D., Hirose Y., Kobayashi T., Matsuda Y., Wakai T. (2014). Sphingosine-1-phosphate transporters as targets for cancer therapy. BioMed Res. Int..

[37] Liu S., Paknejad N., Zhu L., Kihara Y., Ray M., Chun J., Liu W., Hite R.K., Huang X.Y. (2022). Differential activation mechanisms of lipid GPCRs by lysophosphatidic acid and sphingosine 1-phosphate. Nat. Commun..

[38] Yu L., He L., Gan B., Ti R., Xiao Q., Hu H., Zhu L., Wang S., Ren R. (2022). Structural insights into sphingosine-1-phosphate receptor activation. Proc. Natl. Acad. Sci. USA.

[39] Taniguchi R., Inoue A., Sayama M., Uwamizu A., Yamashita K., Hirata K., Yoshida M., Tanaka Y., Kato H.E., Nakada-Nakura Y. (2017). Structural insights into ligand recognition by the lysophosphatidic acid receptor LPA6. Nature.

[40] Kalari S., Zhao Y., Spannhake E.W., Berdyshev E.V., Natarajan V. (2009). Role of acylglycerol kinase in LPA-induced IL-8 secretion and transactivation of epidermal growth factor-receptor in human bronchial epithelial cells. Am. J. Physiol. Lung Cell. Mol. Physiol..

[41] Uehata M., Ishizaki T., Satoh H., Ono T., Kawahara T., Morishita T., Tamakawa H., Yamagami K., Inui J., Maekawa M. (1997). Calcium sensitization of smooth muscle mediated by a Rho-associated protein kinase in hypertension. Nature.

[42] Kume H. (2008). RhoA/Rho-kinase as a therapeutic target in asthma. Curr. Med. Chem..

[43] Dacheux M.A., Norman D.D., Tigyi G.J., Lee S.C. (2023). Emerging roles of lysophosphatidic acid receptor subtype 5 (LPAR5) in inflammatory diseases and cancer. Pharmacol. Ther..

[44] Martin L.D., Rochelle L.G., Fischer B.M., Krunkosky T.M., Adler K.B. (1997). Airway epithelium as an effector of inflammation: Molecular regulation of secondary mediators. Eur. Respir. J..

[45] Zhao Y., Natarajan V. (2009). Lysophosphatidic acid signaling in airway epithelium: Role in airway inflammation and remodeling. Cell. Signal..

[46] Wang L., Cummings R., Zhao Y., Kazlauskas A., Sham J.K., Morris A., Georas S., Brindley D.N., Natarajan V. (2003). Involvement of phospholipase D2 in lysophosphatidate-induced transactivation of platelet-derived growth factor receptor-beta in human bronchial epithelial cells. J. Biol. Chem..

[47] Georas S.N., Berdyshev E., Hubbard W., Gorshkova I.A., Usatyuk P.V., Saatian B., Myers A.C., Williams M.A., Xiao H.Q., Liu M. (2007). Lysophosphatidic acid is detectable in human bronchoalveolar lavage fluids at baseline and increased after segmental allergen challenge. Clin. Exp. Allergy.

[48] Zhao Y., Usatyuk P.V., Cummings R., Saatian B., He D., Watkins T., Morris A., Spannhake E.W., Brindley D.N., Natarajan V. (2005). Lipid phosphate phosphatase-1 regulates lysophosphatidic acid-induced calcium release, NF-kB activation and interleukin-8 secretion in human bronchial epithelial cells. Biochem. J..

[49] He D., Natarajan V., Stern R., Gorshkova I.A., Solway J., Spannhake E.W., Zhao Y. (2008). Lysophosphatidic acid-induced transactivation of epidermal growth factor receptor regulates cyclo-oxygenase-2 expression and prostaglandin E(2) release via C/EBPbeta in human bronchial epithelial cells. Biochem. J..

[50] Zhao Y., He D., Zhao J., Wang L., Leff A.R., Spannhake E.W., Georas S., Natarajan V. (2007). Lysophosphatidic acid induces interleukin-13 (IL-13) receptor alpha2 expression and inhibits IL-13 signaling in primary human bronchial epithelial cells. J. Biol. Chem..

[51] Medoff B.D., Landry A.L., Wittbold K.A., Sandall B.P., Derby M.C., Cao Z., Adams J.C., Xavier R.J. (2009). CARMA3 mediates lysophosphatidic acid-stimulated cytokine secretion by bronchial epithelial cells. Am. J. Respir. Cell Mol. Biol..

[52] Zhao J., He D., Berdyshev E., Zhong M., Salgia R., Morris A.J., Smyth S.S., Natarajan V., Zhao Y. (2011). Autotaxin induces lung epithelial cell migration through lysoPLD activity-dependent and -independent pathways. Biochem. J..

[53] Ren Y., Guo L., Tang X., Apparsundaram S., Kitson C., Deguzman J., Fuentes M.E., Coyle L., Majmudar R., Allard J. (2013). Comparing the differential effects of LPA on the barrier function of human pulmonary endothelial cells. Microvasc. Res..

[54] Cai J., Wei J., Li S., Suber T., Zhao J. (2017). AM966, an Antagonist of Lysophosphatidic Acid Receptor 1, Increases Lung Microvascular Endothelial Permeability through Activation of Rho Signaling Pathway and Phosphorylation of VE-Cadherin. Mediat. Inflamm..

[55] Shlyonsky V., Naeije R., Mies F. (2014). Possible role of lysophosphatidic acid in rat model of hypoxic pulmonary vascular remodeling. Pulm. Circ..

[56] Hashimoto T., Yamashita M., Ohata H., Momose K. (2003). Lysophosphatidic acid enhances in vivo infiltration and activation of guinea pig eosinophils and neutrophils via a Rho/Rho-associated protein kinase-mediated pathway. J. Pharmacol. Sci..

[57] Itagaki K., Kannan K.B., Hauser C.J. (2005). Lysophosphatidic acid triggers calcium entry through a non-store-operated pathway in human neutrophils. J. Leukoc. Biol..

[58] Rahaman M., Costello R.W., Belmonte K.E., Gendy S.S., Walsh M.T. (2006). Neutrophil sphingosine 1-phosphate and lysophosphatidic acid receptors in pneumonia. Rahaman M, Costello RW, Belmonte KE, Gendy SS, Walsh MT. Am. J. Respir. Cell Mol. Biol..

[59] Miyabe C., Miyabe Y., Nagai J., Miura N.N., Ohno N., Chun J., Tsuboi R., Ueda H., Miyasaka M., Miyasaka N. (2019). Abrogation of lysophosphatidic acid receptor 1 ameliorates murine vasculitis. Arthritis Res. Ther..

[60] Idzko M., Laut M., Panther E., Sorichter S., Dürk T., Fluhr J.W., Herouy Y., Mockenhaupt M., Myrtek D., Elsner P. (2004). Lysophosphatidic acid induces chemotaxis, oxygen radical production, CD11b up-regulation, Ca^2+^ mobilization, and actin reorganization in human eosinophils via pertussis toxin-sensitive G proteins. J. Immunol..

[61] Bagga S., Price K.S., Lin D.A., Friend D.S., Austen K.F., Boyce J.A. (2004). Lysophosphatidic acid accelerates the development of human mast cells. Blood.

[62] Hashimoto T., Ohata H., Honda K. (2006). Lysophosphatidic acid (LPA) induces plasma exudation and histamine release in mice via LPA receptors. J. Pharmacol. Sci..

[63] Lin D.A., Boyce J.A. (2005). IL-4 regulates MEK expression required for lysophosphatidic acid-mediated chemokine generation by human mast cells. J. Immunol..

[64] Toews M.L., Ustinova E.E., Schultz H.D. (1997). Lysophosphatidic acid enhances contractility of isolated airway smooth muscle. J. Appl. Physiol..

[65] Kume H., Takai A., Tokuno H., Tomita T. (1989). Regulation of Ca^2+^-dependent K^+^-channel activity in tracheal myocytes by phosphorylation. Nature.

[66] Kume H., Graziano M.P., Kotlikoff M.I. (1992). Stimulatory and inhibitory regulation of calcium-activated potassium channels by guanine nucleotide-binding proteins. Proc. Natl. Acad. Sci. USA.

[67] Kume H., Hall I.P., Washabau R.J., Takagi K., Kotlikoff M.I. (1994). β-Adrenergic agonists regulate K_Ca_ channels in airway smooth muscle by cAMP-dependent and -independent mechanisms. J. Clin. Investig..

[68] Kume H., Mikawa K., Takagi K., Kotlikoff M.I. (1995). Role of G proteins and K_Ca_ channels in the muscarinic and β-adrenergic regulation of airway smooth muscle. Am. J. Physiol..

[69] Ito S., Kume H., Honjo H., Katoh H., Kodama I., Yamaki K., Hayashi H. (2001). Possible involvement of Rho kinase in Ca^2+^ sensitization and mobilization by MCh in tracheal smooth muscle. Am. J. Physiol. Lung Cell. Mol. Physiol..

[70] Oguma T., Kume H., Ito S., Takeda N., Honjo H., Kodama I., Shimokata K., Kamiya K. (2006). Involvement of reduced sensitivity to Ca^2+^ in β-adrenergic action on airway smooth muscle. Clin. Exp. Allergy.

[71] Kume H., Fukunaga K., Oguma T. (2015). Research and development of bronchodilators for asthma and COPD with a focus on G protein/K_Ca_ channel linkage and β_2_-adrenergic intrinsic efficacy. Pharmacol. Ther..

[72] Kume H., Nishiyama O., Isoya T., Higashimoto Y., Tohda Y., Noda Y. (2018). Involvement of Allosteric Effect and K_Ca_ Channels in Crosstalk between β_2_-Adrenergic and Muscarinic M_2_ Receptors in Airway Smooth Muscle. Int. J. Mol. Sci..

[73] Oguma T., Ito S., Kondo M., Makino Y., Shimokata K., Honjo H., Kamiya K., Kume H. (2007). Roles of P2X receptors and Ca^2+^ sensitization in extracellular adenosine triphosphate-induced hyperresponsiveness in airway smooth muscle. Clin. Exp. Allergy.

[74] Kume H., Takagi K. (1999). Inhibition of β-adrenergic desensitization by K_Ca_ channels in human trachealis. Am. J. Respir. Crit. Care Med..

[75] Kume H., Ishikawa T., Oguma T., Ito S., Shimokata K., Kotlikoff M.I. (2003). Involvement of Ca^2+^ mobilization in tachyphylaxis to β-adrenergic receptors in trachealis. Am. J. Respir. Cell Mol. Biol..

[76] Ikenouchi T., Kume H., Oguma T., Makino Y., Shiraki A., Ito Y., Shimokata K. (2008). Role of Ca^2+^ mobilization in desensitization of β-adrenoceptors by platelet-derived growth factor in airway smooth muscle. Eur. J. Pharmacol..

[77] Kobayashi M., Kume H., Oguma T., Makino Y., Ito Y., Shimokata K. (2008). Mast cell tryptase causes homologous desensitization of β-adrenoceptors by Ca^2+^ sensitization in tracheal smooth muscle. Clin. Exp. Allergy.

[78] Fukunaga K., Kume H., Oguma T., Shigemori W., Tohda Y., Ogawa E., Nakano Y. (2016). Involvement of Ca^2+^ Signaling in the Synergistic Effects between Muscarinic Receptor Antagonists and β₂-Adrenoceptor Agonists in Airway Smooth Muscle. Int. J. Mol. Sci..

[79] Sakai J., Oike M., Hirakawa M., Ito Y. (2003). Theophylline and cAMP inhibit lysophosphatidic acid-induced hyperresponsiveness of bovine tracheal smooth muscle cells. J. Physiol..

[80] Cerutis D.R., Nogami M., Anderson J.L., Churchill J.D., Romberger D.J., Rennard S.I., Toews M.L. (1997). Lysophosphatidic acid and EGF stimulate mitogenesis in human airway smooth muscle cells. Am. J. Physiol..

[81] Ediger T.L., Toews M.L. (2000). Synergistic stimulation of airway smooth muscle cell mitogenesis. J. Pharmacol. Exp. Ther..

[82] Ediger T.L., Danforth B.L., Toews M.L. (2002). Lysophosphatidic acid upregulates the epidermal growth factor receptor in human airway smooth muscle cells. Am. J. Physiol. Lung Cell. Mol. Physiol..

[83] Hirakawa M., Karashima Y., Watanabe M., Kimura C., Ito Y., Oike M. (2007). Protein kinase A inhibits lysophosphatidic acid-induced migration of airway smooth muscle cells. J. Pharmacol. Exp. Ther..

[84] Hirshman C.A., Emala C.W. (1999). Actin reorganization in airway smooth muscle cells involves Gq and Gi-2 activation of Rho. Am. J. Physiol..

[85] Zhao Y., He D., Stern R., Usatyuk P.V., Spannhake E.W., Salgia R., Natarajan V. (2007). Lysophosphatidic acid modulates c-Met redistribution and hepatocyte growth factor/c-Met signaling in human bronchial epithelial cells through PKC delta and E-cadherin. Cell. Signal..

[86] Zhao Y., Zhao J., Mialki R.K., Wei J., Spannhake E.W., Salgia R., Natarajan V. (2013). Lipopolysaccharide-induced phosphorylation of c-Met tyrosine residue 1003 regulates c-Met intracellular trafficking and lung epithelial barrier function. Am. J. Physiol. Lung Cell. Mol. Physiol..

[87] Xiao C., Puddicombe S.M., Field S., Haywood J., Broughton-Head V., Puxeddu I., Haitchi H.M., Vernon-Wilson E., Sammut D., Bedke N. (2011). Defective epithelial barrier function in asthma. J. Allergy Clin. Immunol..

[88] Park G.Y., Lee Y.G., Berdyshev E., Nyenhuis S., Du J., Fu P., Gorshkova I.A., Li Y., Chung S., Karpurapu M. (2013). Autotaxin production of lysophosphatidic acid mediates allergic asthmatic inflammation. Am. J. Respir. Crit. Care Med..

[89] Kim H.J., Sim M.S., Lee D.H., Kim C., Choi Y., Park H.S., Chung I.Y. (2020). Lysophosphatidylserine induces eosinophil extracellular trap formation and degranulation: Implications in severe asthma. Allergy.

[90] Yoder M., Zhuge Y., Yuan Y., Holian O., Kuo S., van Breemen R., Thomas L.L., Lum H. (2014). Bioactive lysophosphatidylcholine 16:0 and 18:0 are elevated in lungs of asthmatic subjects. Allergy Asthma. Immunol. Res..

[91] Jolly P.S., Rosenfeldt H.M., Milstien S., Spiegel S. (2002). The roles of sphingosine-1-phosphate in asthma. Mol. Immunol..

[92] Liu H., Li L., Chen Z., Song Y., Liu W., Gao G., Li L., Jiang J., Xu C., Yan G. (2021). S1PR_2_ Inhibition Attenuates Allergic Asthma Possibly by Regulating Autophagy. Front. Pharmacol..

[93] Goetzl E.J., Kong Y., Voice J.K. (2000). Cutting edge: Differential constitutive expression of functional receptors for lysophosphatidic acid by human blood lymphocytes. J. Immunol..

[94] Rubenfeld J., Guo J., Sookrung N., Chen R., Chaicumpa W., Casolaro V., Zhao Y., Natarajan V., Georas S. (2006). Lysophosphatidic acid enhances interleukin-13 gene expression and promoter activity in T cells. Am. J. Physiol. Lung Cell. Mol. Physiol..

[95] Emo J., Meednu N., Chapman T.J., Rezaee F., Balys M., Randall T., Rangasamy T., Georas S.N. (2012). LPA_2_ is a negative regulator of both dendritic cell activation and murine models of allergic lung inflammation. J. Immunol..

[96] Gustin C., Van Steenbrugge M., Raes M. (2008). LPA modulates monocyte migration directly and via LPA-stimulated endothelial cells. Am. J. Physiol. Cell Physiol..

[97] Ray R., Rai V. (2017). Lysophosphatidic acid converts monocytes into macrophages in both mice and humans. Blood.

[98] Chang C.L., Lin M.E., Hsu H.Y., Yao C.L., Hwang S.M., Pan C.Y., Hsu C.Y., Lee H. (2008). Lysophosphatidic acid-induced interleukin-1 beta expression is mediated through Gi/Rho and the generation of reactive oxygen species in macrophages. J. Biomed. Sci..

[99] Kume H., Yamada R., Sato Y., Togawa R. (2023). Airway Smooth Muscle Regulated by Oxidative Stress in COPD. Antioxidants.

[100] Lesh R.E., Emala C.W., Lee H.T., Zhu D., Panettieri R.A., Hirshman C.A. (2001). Inhibition of geranylgeranylation blocks agonist-induced actin reorganization in human airway smooth muscle cells. Am. J. Physiol. Lung Cell. Mol. Physiol..

[101] Georas S.N. (2013). Allergic to autotaxin. A new role for lysophospholipase d and lysophosphatidic Acid in asthma?. Am. J. Respir. Crit. Care Med..

[102] Jendzjowsky N.G., Roy A., Iftinca M., Barioni N.O., Kelly M.M., Herrington B.A., Visser F., Altier C., Wilson R.J.A. (2021). PKCε stimulation of TRPV1 orchestrates carotid body responses to asthmakines. J. Physiol..

[103] Kondo M., Tezuka T., Ogawa H., Koyama K., Bando H., Azuma M., Nishioka Y. (2021). Lysophosphatidic Acid Regulates the Differentiation of Th2 Cells and Its Antagonist Suppresses Allergic Airway Inflammation. Int. Arch. Allergy Immunol..

[104] Knowlden S.A., Hillman S.E., Chapman T.J., Patil R., Miller D.D., Tigyi G., Georas S.N. (2016). Novel Inhibitory Effect of a Lysophosphatidic Acid 2 Agonist on Allergen-Driven Airway Inflammation. Am. J. Respir. Cell Mol. Biol..

[105] Georas S.N. (2021). LPA and Autotaxin: Potential Drug Targets in Asthma?. Cell Biochem. Biophys..

[106] Yin H., Kamakura N., Qian Y., Tatsumi M., Ikuta T., Liang J., Xu Z., Xia R., Zhang A., Guo C. (2024). Insights into lysophosphatidylserine recognition and Gα_12/13_-coupling specificity of P2Y10. Cell Chem. Biol..

[107] Sim M.S., Kim H.J., Jo S.H., Kim C., Chung I.Y. (2022). Lysophosphatidylserine Induces MUC5AC Production via the Feedforward Regulation of the TACE-EGFR-ERK Pathway in Airway Epithelial Cells in a Receptor-Independent Manner. Int. J. Mol. Sci..

[108] Park K.S., Lee H.Y., Kim M.K., Shin E.H., Jo S.H., Kim S.D., Im D.S., Bae Y.S. (2006). Lysophosphatidylserine stimulates L2071 mouse fibroblast chemotactic migration via a process involving pertussis toxin-sensitive trimeric G-proteins. Mol. Pharmacol..

[109] Sugo T., Tachimoto H., Chikatsu T., Murakami Y., Kikukawa Y., Sato S., Kikuchi K., Nagi T., Harada M., Ogi K. (2006). Identification of a lysophosphatidylserine receptor on mast cells. Biochem. Biophys. Res. Commun..

[110] Kawamoto K., Aoki J., Tanaka A., Itakura A., Hosono H., Arai H., Kiso Y., Matsuda H. (2002). Nerve growth factor activates mast cells through the collaborative interaction with lysophosphatidylserine expressed on the membrane surface of activated platelets. J. Immunol..

[111] Barnes M.J., Li C.M., Xu Y., An J., Huang Y., Cyster J.G. (2015). The lysophosphatidylserine receptor GPR174 constrains regulatory T cell development and function. J. Exp. Med..

[112] Kume N., Cybulsky M.I., Gimbrone M.A. (1992). Lysophosphatidylcholine, a component of atherogenic lipoproteins, induces mononuclear leukocyte adhesion molecules in cultured human and rabbit arterial endothelial cells. J. Clin. Investig..

[113] Kume N., Gimbrone M.A. (1994). Lysophosphatidylcholine transcriptionally induces growth factor gene expression in cultured human endothelial cells. J. Clin. Investig..

[114] Zhang Q., Zhang W., Liu J., Yang H., Hu Y., Zhang M., Bai T., Chang F. (2021). Lysophosphatidylcholine promotes intercellular adhesion molecule-1 and vascular cell adhesion molecule-1 expression in human umbilical vein endothelial cells via an orphan G protein receptor 2-mediated signaling pathway. Bioengineered.

[115] Jing Q., Xin S.M., Zhang W.B., Wang P., Qin Y.W., Pei G. (2000). Lysophosphatidylcholine activates p38 and p42/44 mitogen-activated protein kinases in monocytic THP-1 cells, but only p38 activation is involved in its stimulated chemotaxis. Circ. Res..

[116] Yamamoto N., Homma S. (1991). Vitamin D3 binding protein (group-specific component) is a precursor for the macrophage-activating signal factor from lysophosphatidylcholine-treated lymphocytes. Proc. Natl. Acad. Sci. USA.

[117] Lusis A.J. (2000). Atherosclerosis. Nature.

[118] Matsumoto T., Kobayashi T., Kamata K. (2007). Role of lysophosphatidylcholine (LPC) in atherosclerosis. Curr. Med. Chem..

[119] Asaoka Y., Oka M., Yoshida K., Sasaki Y., Nishizuka Y. (1992). Role of lysophosphatidylcholine in T-lymphocyte activation: Involvement of phospholipase A2 in signal transduction through protein kinase C. Proc. Natl. Acad. Sci. USA.

[120] Suenaga H., Kamata K. (1999). Marked dissociation between intracellular Ca^2+^ level and contraction on exposure of rat aorta to lysophosphatidylcholine. Eur. J. Pharmacol..

[121] Ding W.G., Toyoda F., Ueyama H., Matsuura H. (2011). Lysophosphatidylcholine enhances IKs currents in cardiac myocytes through activation of G protein, PKC and Rho signaling pathways. J. Mol. Cell. Cardiol..

[122] Law S.H., Chan M.L., Marathe G.K., Parveen F., Chen C.H., Ke L.Y. (2019). An Updated Review of Lysophosphatidylcholine Metabolism in Human Diseases. Int. J. Mol. Sci..

[123] Zhuge Y., Yuan Y., van Breemen R., Degrand M., Holian O., Yoder M., Lum H. (2014). Stimulated bronchial epithelial cells release bioactive lysophosphatidylcholine 16:0, 18:0, and 18:1. Allergy Asthma. Immunol. Res..

[124] Feldman C., Anderson R., Theron A.J., Steel H.C., van Rensburg C.E., Cole P.J., Wilson R. (2001). Vitamin E attenuates the injurious effects of bioactive phospholipids on human ciliated epithelium in vitro. Eur. Respir. J..

[125] Murugesan G., Sandhya Rani M.R., Gerber C.E., Mukhopadhyay C., Ransohoff R.M., Chisolm G.M., Kottke-Marchant K. (2003). Lysophosphatidylcholine regulates human microvascular endothelial cell expression of chemokines. J. Mol. Cell. Cardiol..

[126] Murugesan G., Fox P.L. (1996). Role of lysophosphatidylcholine in the inhibition of endothelial cell motility by oxidized low density lipoprotein. J. Clin. Investig..

[127] Nakano T., Raines E.W., Abraham J.A., Klagsbrun M., Ross R. (1994). Lysophosphatidylcholine upregulates the level of heparin-binding epidermal growth factor-like growth factor mRNA in human monocytes. Proc. Natl. Acad. Sci. USA.

[128] Weber C., Kitayama J., Springer T.A. (1996). Differential regulation of β1 and β2 integrin avidity by chemoattractants in eosinophils. Proc. Natl. Acad. Sci. USA.

[129] Zhu X., Learoyd J., Butt S., Zhu L., Usatyuk P.V., Natarajan V., Munoz N.M., Leff A.R. (2007). Regulation of eosinophil adhesion by lysophosphatidylcholine via a non-store-operated Ca^2+^ channel. Am. J. Respir. Cell Mol Biol..

[130] Zou Y., Kim C.H., Chung J.H., Kim J.Y., Chung S.W., Kim M.K., Im D.S., Lee J., Yu B.P., Chung H.Y. (2007). Upregulation of endothelial adhesion molecules by lysophosphatidylcholine. Involvement of G protein-coupled receptor GPR4. FEBS J..

[131] Kugiyama K., Kerns S.A., Morrisett J.D., Roberts R., Henry P.D. (1990). Impairment of endothelium-dependent arterial relation by lysolecithin in modified low-density lipoproteins. Nature.

[132] Galle J., Mameghani A., Bolz S.S., Gambaryan S., Görg M., Quaschning T., Raff U., Barth H., Seibold S., Wanner C. (2003). Oxidized LDL and its compound lysophosphatidylcholine potentiate AngII-induced vasoconstriction by stimulation of RhoA. J. Am. Soc. Nephrol..

[133] Kume H., Takagi K. (1997). Inhibitory effects of Gs on desensitization of β-adrenergic receptors in tracheal smooth muscle. Am. J. Physiol..

[134] Bansal P., Gaur S.N., Arora N. (2016). Lysophosphatidylcholine plays critical role in allergic airway disease manifestation. Sci. Rep..

[135] Nobata K., Kurashima K., Fujimura M., Abo M., Ishiura Y., Kasahara K., Nakao S. (2005). Inhaled lysophosphatidylcholine provokes bronchoconstriction in guinea pigs in vivo. Eur. J. Pharmacol..

[136] Mehta D., Gupta S., Gaur S.N., Gangal S.V., Agrawal K.P. (1990). Increased leukocyte phospholipase A2 activity and plasma lysophosphatidylcholine levels in asthma and rhinitis and their relationship to airway sensitivity to histamine. Am. Rev. Respir. Dis..

[137] Kume H., Hojo M., Hashimoto N. (2019). Eosinophil Inflammation and Hyperresponsiveness in the Airways as Phenotypes of COPD, and Usefulness of Inhaled Glucocorticosteroids. Front. Pharmacol..

[138] Rice K.L., Duane P.G., Niewoehner D.E. (1987). Lysophosphatidylcholine augments elastase-induced alveolar epithelial permeability and emphysema in the hamster. Am. Rev. Respir. Dis..

[139] Tanosaki T., Mikami Y., Shindou H., Suzuki T., Hashidate-Yoshida T., Hosoki K., Kagawa S., Miyata J., Kabata H., Masaki K. (2022). Lysophosphatidylcholine Acyltransferase 1 Deficiency Promotes Pulmonary Emphysema via Apoptosis of Alveolar Epithelial Cells. Inflammation.

[140] Okamoto H., Takuwa N., Gonda K., Okazaki H., Chang K., Yatomi Y., Shigematsu H., Takuwa Y. (1998). EDG1 is a functional sphingosine-1-phosphate receptor that is linked via a Gi/o to multiple signaling pathways, including phospholipase C activation, Ca^2+^ mobilization, Ras-mitogen-activated protein kinase activation, and adenylate cyclase inhibition. J. Biol. Chem..

[141] Ishii I., Ye X., Friedman B., Kawamura S., Contos J.J., Kingsbury M.A., Yang A.H., Zhang G., Brown J.H., Chun J. (2002). Marked perinatal lethality and cellular signaling deficits in mice null for the two sphingosine 1-phosphate (S1P) receptors, S1P_2_/LP_B2_/EDG-5 and S1P_3_/LP_B3_/EDG-3. J. Biol. Chem..

[142] Sadahira Y., Ruan F., Hakomori S., Igarashi Y. (1992). Sphingosine 1-phosphate, a specific endogenous signaling molecule controlling cell motility and tumor cell invasiveness. Proc. Natl. Acad. Sci. USA.

[143] Garcia J.G., Liu F., Verin A.D., Birukova A., Dechert M.A., Gerthoffer W.T., Bamberg J.R., English D. (2001). Sphingosine 1-phosphate promotes endothelial cell barrier integrity by Edg-dependent cytoskeletal rearrangement. J. Clin. Investig..

[144] Xiong Y., Yang P., Proia R.L., Hla T. (2014). Erythrocyte-derived sphingosine 1-phosphate is essential for vascular development. J. Clin. Investig..

[145] Venkataraman K., Thangada S., Michaud J., Oo M.L., Ai Y., Lee Y.M., Wu M., Parikh N.S., Khan F., Proia R.L. (2006). Extracellular export of sphingosine kinase-1a contributes to the vascular S1P gradient. Biochem. J..

[146] Olivera A., Allende M.L., Proia R.L. (2013). Shaping the landscape: Metabolic regulation of S1P gradients. Biochim. Biophys. Acta.

[147] Proia R.L., Hla T. (2015). Emerging biology of sphingosine-1-phosphate: Its role in pathogenesis and therapy. J. Clin. Investig..

[148] Ebenezer D.L., Fu P., Natarajan V. (2016). Targeting sphingosine-1-phosphate signaling in lung diseases. Pharmacol. Ther..

[149] Camerer E., Regard J.B., Cornelissen I., Srinivasan Y., Duong D.N., Palmer D., Pham T.H., Wong J.S., Pappu R., Coughlin S.R. (2009). Sphingosine-1-phosphate in the plasma compartment regulates basal and inflammation-induced vascular leak in mice. J. Clin. Investig..

[150] Knipe R.S., Spinney J.J., Abe E.A., Probst C.K., Franklin A., Logue A., Giacona F., Drummond M., Griffith J., Brazee P.L. (2022). Endothelial-Specific Loss of Sphingosine-1-Phosphate Receptor 1 Increases Vascular Permeability and ExacerbatesBleomycin-induced Pulmonary Fibrosis. Am. J. Respir. Cell Mol. Biol..

[151] Karam M., Auclair C. (2023). Sphingosine-1-Phosphate as Lung and Cardiac Vasculature Protecting Agent in SARS-CoV-2 Infection. Int. J. Mol. Sci..

[152] Marfia G., Navone S., Guarnaccia L., Campanella R., Mondoni M., Locatelli M., Barassi A., Fontana L., Palumbo F., Garzia E. (2021). Decreased serum level of sphingosine-1-phosphate: A novel predictor of clinical severity in COVID-19. EMBO Mol. Med..

[153] Olivera A., Eisner C., Kitamura Y., Dillahunt S., Allende L., Tuymetova G., Watford W., Meylan F., Diesner S.C., Li L. (2010). Sphingosine kinase 1 and sphingosine-1-phosphate receptor 2 are vital to recovery from anaphylactic shock in mice. J. Clin. Investig..

[154] Goel K., Beatman E.L., Egersdorf N., Scruggs A., Cao D., Berdyshev E.V., Schweitzer K.S., Petrache I. (2021). Sphingosine 1 Phosphate (S1P) Receptor 1 Is Decreased in Human Lung Microvascular Endothelial Cells of Smokers and Mediates S1P Effect on Autophagy. Cells.

[155] Ito S., Suki B., Kume H., Numaguchi Y., Ishii M., Iwaki M., Kondo M., Naruse K., Hasegawa Y., Sokabe M. (2010). Actin cytoskeleton regulates stretch-activated Ca^2+^ influx in human pulmonary microvascular endothelial cells. Am. J. Respir. Cell Mol. Biol..

[156] Choi O.H., Kim J.H., Kinet J.P. (1996). Calcium mobilization via sphingosine kinase in signalling by the Fc epsilon RI antigen receptor. Nature.

[157] Jo H., Shim K., Jeoung D. (2022). The Crosstalk between FcεRI and Sphingosine Signaling in Allergic Inflammation. Int. J. Mol. Sci..

[158] Roviezzo F., Del Galdo F., Abbate G., Bucci M., D’Agostino B., Antunes E., De Dominicis G., Parente L., Rossi F., Cirino G. (2004). Human eosinophil chemotaxis and selective in vivo recruitment by sphingosine 1-phosphate. Proc. Natl. Acad. Sci. USA.

[159] Kimura T., Tomura H., Mogi C., Kuwabara A., Ishiwara M., Shibasawa K., Sato K., Ohwada S., Im D.S., Kurose H. (2006). Sphingosine 1-phosphate receptors mediate stimulatory and inhibitory signalings for expression of adhesion molecules in endothelial cells. Cell. Signal..

[160] Muraki K., Imaizumi Y. (2001). A novel function of sphingosine-1-phosphate to activate a non-selective cation channel in human endothelial cells. J. Physiol..

[161] James B.N., Weigel C., Green C.D., Brown R.D.R., Palladino E.N.D., Tharakan A., Milstien S., Proia R.L., Martin R.K., Spiegel S. (2023). Neutrophilia in severe asthma is reduced in Ormdl3 overexpressing mice. FASEB J..

[162] Rosenfeldt H.M., Amrani Y., Watterson K.R., Murthy K.S., Panettieri R.A., Spiegel S. (2003). Sphingosine-1-phosphate stimulates contraction of human airway smooth muscle cells. FASEB J..

[163] Liu L., Zhai C., Pan Y., Zhu Y., Shi W., Wang J., Yan X., Su X., Song Y., Gao L. (2018). Sphingosine-1-phosphate induces airway smooth muscle cell proliferation, migration, and contraction by modulating Hippo signaling effector YAP. Am. J. Physiol. Lung Cell. Mol. Physiol..

[164] Maguire T.J.A., Yung S., Ortiz-Zapater E., Kayode O.S., Till S., Corrigan C., Siew L.Q.C., Knock G.A., Woszczek G. (2023). Sphingosine-1-phosphate induces airway smooth muscle hyperresponsiveness and proliferation. J. Allergy Clin. Immunol..

[165] Roviezzo F., Di Lorenzo A., Bucci M., Brancaleone V., Vellecco V., De Nardo M., Orlotti D., De Palma R., Rossi F., D’Agostino B. (2007). Sphingosine-1-phosphate/sphingosine kinase pathway is involved in mouse airway hyperresponsiveness. Am. J. Respir. Cell Mol. Biol..

[166] Makino Y., Kume H., Oguma T., Sugishita M., Shiraki A., Hasegawa Y., Honjo H., Kamiya K. (2012). Role of sphingosine-1-phosphate in β-adrenoceptor desensitization via Ca^2+^ sensitization in airway smooth muscle. Allergol. Int..

[167] Rumzhum N.N., Rahman M.M., Oliver B.G., Ammit A.J. (2016). Effect of sphingosine 1-phosphate on cyclo-oxygenase-2 expression, prostaglandin E2 secretion, and β_2_-adrenergic receptor desensitization. Am. J. Respir. Cell Mol. Biol..

[168] Ishikawa T., Kume H., Kondo M., Ito Y., Yamaki K., Shimokata K. (2003). Inhibitory effects of interferon-γ on the heterologous desensitization of β-adrenoceptors by transforming growth factor-β_1_ in tracheal smooth muscle. Clin. Exp. Allergy.

[169] Idzko M., Hammad H., van Nimwegen M., Kool M., Müller T., Soullié T., Willart M.A., Hijdra D., Hoogsteden H.C., Lambrecht B.N. (2006). Local application of FTY720 to the lung abrogates experimental asthma by altering dendritic cell function. J. Clin. Investig..

[170] Edmonds Y., Milstien S., Spiegel S. (2011). Development of small-molecule inhibitors of sphingosine-1-phosphate signaling. Pharmacol. Ther..

[171] Karmouty-Quintana H., Siddiqui S., Hassan M., Tsuchiya K., Risse P.A., Xicota-Vila L., Marti-Solano M., Martin J.G. (2012). Treatment with a sphingosine-1-phosphate analog inhibits airway remodeling following repeated allergen exposure. Am. J. Physiol. Lung Cell. Mol. Physiol..

[172] Ammit A.J., Hastie A.T., Edsall L.C., Hoffman R.K., Amrani Y., Krymskaya V.P., Kane S.A., Peters S.P., Penn R.B., Spiegel S. (2001). Sphingosine 1-phosphate modulates human airway smooth muscle cell functions that promote inflammation and airway remodeling in asthma. FASEB J..

[173] Roviezzo F., Sorrentino R., Bertolino A., De Gruttola L., Terlizzi M., Pinto A., Napolitano M., Castello G., D’Agostino B., Ianaro A. (2015). S1P-induced airway smooth muscle hyperresponsiveness and lung inflammation in vivo: Molecular and cellular mechanisms. Br. J. Pharmacol..

[174] Patil M.J., Meeker S., Bautista D., Dong X., Undem B.J. (2019). Sphingosine-1-phosphate activates mouse vagal airway afferent C-fibres via S1PR3 receptors. J. Physiol..

[175] Trifilieff A., Fozard J.R. (2012). Sphingosine-1-phosphate-induced airway hyper-reactivity in rodents is mediated by the sphingosine-1-phosphate type 3 receptor. J. Pharmacol. Exp. Ther..

[176] Price M.M., Oskeritzian C.A., Falanga Y.T., Harikumar K.B., Allegood J.C., Alvarez S.E., Conrad D., Ryan J.J., Milstien S., Spiegel S. (2013). A specific sphingosine kinase 1 inhibitor attenuates airway hyperresponsiveness and inflammation in a mast cell-dependent murine model of allergic asthma. J. Allergy Clin. Immunol..

[177] Fuerst E., Foster H.R., Ward J.P., Corrigan C.J., Cousins D.J., Woszczek G. (2014). Sphingosine-1-phosphate induces pro-remodelling response in airway smooth muscle cells. Allergy.

[178] Moffatt M.F., Kabesch M., Liang L., Dixon A.L., Strachan D., Heath S., Depner M., von Berg A., Bufe A., Rietschel E. (2007). Genetic variants regulating ORMDL3 expression contribute to the risk of childhood asthma. Nature.

[179] Galanter J., Choudhry S., Eng C., Nazario S., Rodríguez-Santana J.R., Casal J., Torres-Palacios A., Salas J., Chapela R., Watson H.G. (2008). ORMDL3 gene is associated with asthma in three ethnically diverse populations. Am. J. Respir. Crit. Care Med..

[180] Ntontsi P., Photiades A., Zervas E., Xanthou G., Samitas K. (2021). Genetics and Epigenetics in Asthma. Int. J. Mol. Sci..

[181] Ono J.G., Kim B.I., Zhao Y., Christos P.J., Tesfaigzi Y., Worgall T.S., Worgall S. (2020). Decreased sphingolipid synthesis in children with 17q21 asthma-risk genotypes. J. Clin. Investig..

[182] Calışkan M., Bochkov Y.A., Kreiner-Møller E., Bønnelykke K., Stein M.M., Du G., Bisgaard H., Jackson D.J., Gern J.E., Lemanske R.F. (2013). Rhinovirus wheezing illness and genetic risk of childhood-onset asthma. N. Engl. J. Med..

[183] Wasserman E., Gomi R., Sharma A., Hong S., Bareja R., Gu J., Balaji U., Veerappan A., Kim B.I., Wu W. (2022). Human Rhinovirus Infection of the Respiratory Tract Affects Sphingolipid Synthesis. Am. J. Respir. Cell Mol. Biol..

[184] Worgall T.S., Veerappan A., Sung B., Kim B.I., Weiner E., Bholah R., Silver R.B., Jiang X.C., Worgall S. (2013). Impaired sphingolipid synthesis in the respiratory tract induces airway hyperreactivity. Sci. Transl. Med..

[185] Yoshida K., Morishima Y., Ano S., Sakurai H., Kuramoto K., Tsunoda Y., Yazaki K., Nakajima M., Sherpa M.T., Matsuyama M. (2023). ELOVL6 deficiency aggravates allergic airway inflammation through the ceramide-S1P pathway in mice. J. Allergy Clin. Immunol..

[186] Yoshida K., Morishima Y., Ishii Y., Mastuzaka T., Shimano H., Hizawa N. (2024). Abnormal saturated fatty acids and sphingolipids metabolism in asthma. Respir. Investig..

[187] Yaginuma S., Omi J., Kano K., Aoki J. (2023). Lysophospholipids and their producing enzymes: Their pathological roles and potential as pathological biomarkers. Pharmacol. Ther..

[188] Nolin J.D., Lai Y., Ogden H.L., Manicone A.M., Murphy R.C., An D., Frevert C.W., Ghomashchi F., Naika G.S., Gelb M.H. (2017). Secreted PLA2 group X orchestrates innate and adaptive immune responses to inhaled allergen. JCI Insight.

